# Optimal control and the dynamics of ancestral lineages

**DOI:** 10.1093/genetics/iyag122

**Published:** 2026-05-18

**Authors:** Colin LaMont

**Affiliations:** Precision Immunotherapy Alliance (PRIMA), Institute of Clinical Medicine, University of Oslo, Problemveien 11, Oslo 0371, Norway; Department of Cancer Immunology, Institute for Cancer Research, Oslo University Hospital, Montebello, Oslo 0310, Norway; Institute for Biological Physics, University of Cologne, Zülpicher Straße 77a, Cologne 50937, Germany

**Keywords:** reproductive value, ancestral lineages, optimal control, phylogenetic inference, time-varying selection

## Abstract

In resource-constrained populations, the growth rate of an allele is coupled to the frequency of its competitors. As these frequencies are unobserved, exact inference of evolutionary parameters demands integrating over the full ensemble of possible frequency histories. This high-dimensional path integral is computationally prohibitive, and standard phylogenetic approaches avoid its evaluation by treating lineages as unconstrained birth–death processes. However, ignoring the carrying capacity comes at a cost: it necessitates an abundance of free parameters to describe simple allelic replicator dynamics and implicitly assumes an incorrect drift model. We propose a new approach by defining the *fitness potential* (*Ψ*)—a dynamical quantity formally equivalent to the logarithm of reproductive value. By exploiting a duality between evolutionary dynamics and stochastic optimal control, we show that *Ψ* allows for the analytic marginalization of the unknown population history. This transformation yields a tractable likelihood function that strictly enforces competitive constraints while accounting for the information loss due to genetic drift in finite populations. We demonstrate that this framework enables parsimonious, site-specific inference of selection coefficients from phylogenetic trees. This dual-control framework shows ancestral lineages behave as optimal agents: they do not simply climb local fitness gradients, but “anticipate” future selection by navigating a global landscape of reproductive value.

## Teaser

We simulate evolution to solve control problems. Can we also use control theory to better understand biological evolution? We analyzed how ancestral lineages navigate resource-constrained environments. LaMont discovered a mathematical duality: tracing the ancestors of a living population backward in time is formally identical to solving a stochastic optimal control problem. Because of retrospective survivorship bias, ancestral lineages appear to “anticipate” future selection, exactly like rational agents optimizing a path. By exploiting this equivalence, the author developed a practical framework that bypasses computational bottlenecks, allowing researchers to efficiently infer time-varying selection pressures directly from phylogenetic trees.

The fitness landscape is a foundational concept in evolutionary genetics, mapping genotypes to their reproductive success. However, in any finite environment, success is relative: an allele’s growth is constrained by competition with the rest of the population. Birth and death rates of a specific genotype *g* (denoted $\beta_{g}$ and $\delta_{g}$) are linked not to absolute fitness $f_{g}$, but to the fitness relative to the population mean, $\bar{\;f}(x)=f\cdot x$. Consequently, calculating the likelihood for fitness parameters requires continuous knowledge of this mean fitness term, which depends on the frequency of every allele in the population state x. Capturing the rich physics of evolution (for example, clonal interference and competitive exclusion) therefore requires integrating over the full ensemble of possible population histories, i.e. evaluating a path integral over the trajectory of allele frequencies x(t). This task faces a severe curse of dimensionality. As the number of genetic variants or time points increases, the space of possible population trajectories grows exponentially, making the exact path integral computationally prohibitive.

To achieve tractability, standard phylogenetic approaches (like birth–death skyline models Stadler et al. 2013; Bouckaert et al. 2014) avoid this high-dimensional integration by decoupling the lineages. They treat each lineage’s growth rate as an independent, piecewise-constant parameter to be inferred, effectively ignoring the zero-sum constraint that couples lineage growth to the population state x(t). This avoids the need to condition on a population history, but comes at the cost of model parsimony: it requires an explosion of free parameters to describe dynamics that are physically generated by a small number of selection coefficients acting within the governing constraint of the carrying capacity.

## Introduction

We propose a framework for the selective pressure on lineages that respects a finite carrying capacity, enabling parsimonious inference in these biologically relevant regimes. To achieve this, we exploit a formal duality with stochastic optimal control theory, drawing inspiration from methods where log-ratios are used to measure the value of a state. We propose a similar quantity for population genetics to measure the reproductive value of an allele Fisher (1999), Goodman (1982), Grafen (2006) and Barton and Etheridge (2011). This quantity, the fitness potential $\Psi_{g}(x)$, is defined as the log-ratio of an allele *g*’s frequency in the ancestral pool, $w_{g}(x)$, relative to its frequency in the contemporaneous living population, $x_{g}$:

(1) $$\Psi_{g}(x)=\log\;\frac{w_{g}(x)}{x_{g}}$$

Intuitively, this potential measures the enrichment of an allele among ancestors relative to the population in which they lived.

We derive the backward-in-time dynamics of this quantity from standard population-genetic principles. A key result of our analysis is that, in the weak-selection limit, this potential approximately decomposes into a frequency-independent component specific to each allele ($\psi_{g}$) and an allele-independent component shared by all possible lineages (ϕ(x)):

(2) $$\Psi_{g}(x)\approx \psi_{g}-\phi (x)$$

This decomposition is the mechanism that allows tractable inference. By isolating the complex, frequency-dependent population dynamics within a shared term (ϕ(x)), we can effectively marginalize out these unobservable histories. The relevant information for comparative success is retained in the much simpler, allele-specific term ($\psi_{g}$), which is governed by an ordinary differential equation (ODE) rather than a curse-of-dimensionality-afflicted partial differential equation (PDE).

We explore the computational and conceptual implications of this simplification. First, we show how this formalism provides a new lens on adaptation in dynamic environments, revealing a formal duality with optimal control theory. Second, we benchmark the approximation against exact solutions in biallelic systems to establish its valid parameter regimes. Finally, we leverage this validated theory to develop an efficient likelihood framework for inferring time-varying selection directly from phylogenetic trees.

## Methods

### Model setup and definitions

**Fig. 1.**
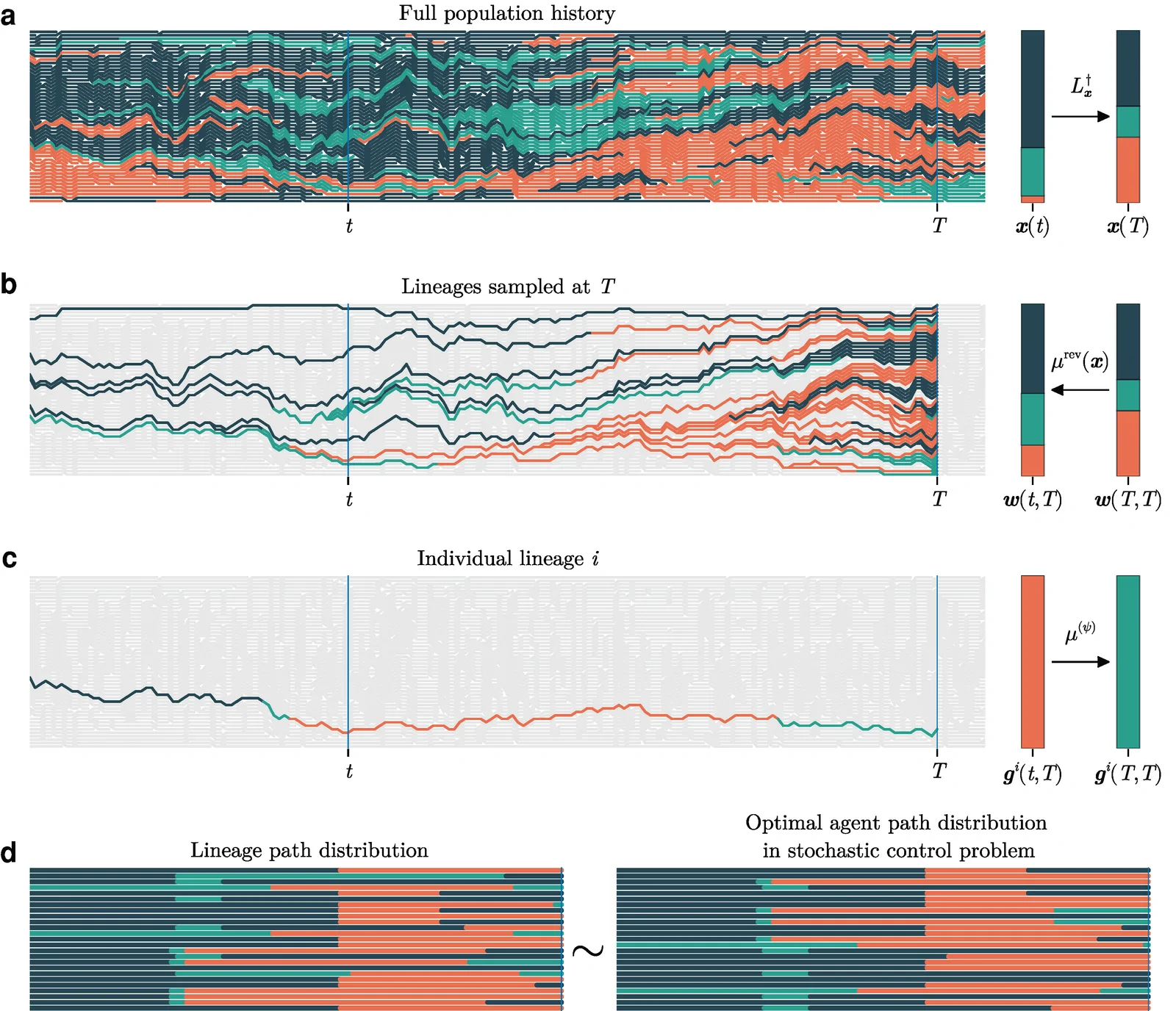
The correspondence between ancestral lineage dynamics and optimal control. a) The full population history evolves forward in time from *t* to *T*, driven by mutation, selection, and drift (mathematically described by the forward Fisher-Kimura generator $L_{x}^{†}$), resulting in the observable population state x(t) b) Conditioning on survival to the sampling time *T* reveals the ancestral lineages (colored paths). The ancestral frequency distribution w(t,T) represents the probability that an ancestor at time *t* has a specific genotype, evolving backward according to the *x*-conditioned operator $\mu^{\mathrm{rev}}(x)$. c) A single surviving lineage *i* traces a discrete path $g^{i}(t,T)$ through genotypic space. While individuals in the full population generate mutations according to *μ*, the surviving lineage appears to follow a biased mutational operator $\mu^{\left(\psi \right)}$, where the bias is driven by the fitness potential *ψ*. d) The distribution of these biological lineage paths (left) is statistically identical to the distribution of optimal agent trajectories in a dual stochastic control problem (right). This duality implies that surviving lineages appear to have “anticipated” future selective pressures by following an optimal control policy.

We model the evolution of a population using the language of a standard Wright-Fisher diffusion or Moran process. Let $x=(x_{1},x_{2},\ldots ,x_{k})$ denote the vector of genotypic frequencies, where $x_{g}$ represents the relative fraction of genotype *g* in the living population such that $\sum x_{g}=1$(Fig. 1, Panel a). This vector encodes the population state, x(t) and evolves forward in time according to a stochastic process driven by mutation (rate matrix *μ*), selection (fitness vector f), and random genetic drift (scaled by population size $N_{e}$). As we consider haploid populations (e.g. viral strains), we use the terms *genotype* and *allele* interchangeably to refer to the specific genetic state of a lineage.

#### The Ancestral Lineage

Our primary goal is to understand the dynamics of a single lineage traced backward from a sampled individual. Unlike the general population, which is shaped by mutation, selection and genetic drift, ancestral lineages have been shaped by an additional factor: the retrospective condition of having successfully left descendants. Consider an experiment where we sample a single individual at the present time *T*. We wish to trace its ancestry backward to time t<T. Let $w_{g}(x,t,T)$ be the probability that the ancestor at time *t* had genotype *g*, given the population state was x at *t*. As depicted in Fig. 1, Panel B, at t=T, the ancestor will be identical to the sampled individual, implying the boundary conditions that w(x,T,T)=x. We derive the dynamics of w(x,t,T) as *t* recedes into the past. We will then perform a change of variables to find the dynamics of the $\Psi_{g}(x,t,T)=\log\;\frac{w_{g}(x,t,T)}{x_{g}(t)}$, our key quantity of interest.

We consider a fixed sampling time *T*, and suppress the *T* dependence in our notation. Furthermore, where the time-dependence *t* is clear from context, we will write $w_{g}(x)$, and $\Psi_{g}(x)$ for brevity.

### Backward evolution of the ancestral frequency

We begin with the dynamics of the ancestral frequencies w(x,t), defined as the probability that an ancestor at time *t* has genotype *g*, given the population state was x. Lineage transport between genotypic states is governed by the frequency-conditioned reversed mutational operator, $\mu^{\mathrm{rev}}$. We derive this by applying Bayes’ rule to the mutation flux. In the forward direction, the total flux of individuals mutating from $g^{\prime }$ to *g* is proportional to the mutation rate $\mu_{g^{\prime }g}$ and the frequency of the source $x_{g^{\prime }}$. The probability that a specific lineage in state *g* originated in $g^{\prime }$ is this flux normalized by the abundance of the destination state $x_{g}$:

(3) $$\mu_{gg^{\prime }}^{\mathrm{rev}}(x)=\mu_{g^{\prime }g}\frac{x_{g^{\prime }}}{x_{g}}\;\text{\textbackslash{},for}\;g\neq g^{\prime }$$

To conserve probability, the diagonal elements are set to the negative sum of the off-diagonals. As we move backward in time, this operator pulls lineages toward historically abundant genotypes. Notably, birth and death events do not appear in this transport term; tracing a surviving lineage backward, coalescent events concentrate the distribution into fewer distinct ancestors, but do not alter the genotype of the lineage itself.

Combining this reversed mutational process with the adjoint Kimura diffusion operator ($L_{x}$) Kimura (1962), which moves the x-conditioning backward in time, we arrive at the full evolution equation:

(4) $$\begin{array}{rl} -\partial_{t}w_{g}(x,t) & =\sum_{g^{\prime }}\left(x_{g}\mu_{gg^{\prime }}\frac{w_{g^{\prime }}(x)}{x_{g^{\prime }}}-x_{g^{\prime }}\mu_{g^{\prime }g}\frac{w_{g}(x)}{x_{g}}\right)\\ & \;+L_{x}w_{g}(x) \end{array}$$

We now perform a change of variables from $w_{g}(x)$ to our core object, the fitness potential $\Psi_{g}(x)=\log\;(w_{g}(x)/x_{g})$. Applying this transformation to the backward dynamics in Equation (4) yields the backward-time evolution for the potential

(5) $$\begin{array}{rl} -\partial_{t}\Psi_{g}(x,t) & =\sum_{g^{\prime }}\left(\mu_{gg^{\prime }}e^{\Psi_{g^{\prime }}(x)-\Psi_{g}(x)}-\frac{x_{g^{\prime }}}{x_{g}}\mu_{g^{\prime }g}\right)\\ & \;+\frac{e^{-\Psi_{g}(x)}}{x_{g}}L_{x}x_{g}e^{\Psi_{g}(x)}. \end{array}$$

Our boundary condition for w is $w_{g}(x,T)=x_{g}$ implies $\Psi_{g}(x,T)=0$.

Expanding this $\frac{x}{w}L_{x}\frac{w}{x}$ sandwich on the selection, mutation, and drift-diffusion operators (see Coordinate transform of the Kimura operator) yields the exact backward evolution for the fitness potential,

(6) $$\begin{array}{rl} -\partial_{t}\Psi_{g}(x,t) & =\sum_{g^{\prime }}\mu_{gg^{\prime }}\left(e^{\Psi_{g^{\prime }}(x)-\Psi_{g}(x)}-1\right)+f_{g}-\sum_{g^{\prime }}x_{g^{\prime }}f_{g^{\prime }}+L_{x}\Psi_{g}(x)\\ & \;+\frac{1}{N_{e}}\left(\frac{\partial \Psi_{g}}{\partial x_{g}}-\sum_{h}x_{h}\frac{\partial \Psi_{g}}{\partial x_{h}}\right)\\ & \;+\frac{1}{2N_{e}}\left[\sum_{h}x_{h}{\left(\frac{\partial \Psi_{g}}{\partial x_{h}}\right)}^{2}-{\left(\sum_{h}x_{h}\frac{\partial \Psi_{g}}{\partial x_{h}}\right)}^{2}\right]. \end{array}$$

#### The forward generator for phylogenetics

This backward-time equation for *Ψ* has a key role in defining the apparent forward mutation rates along a surviving lineage. The apparent forward mutation rates are a central observable quantity in phylogenetics, propagating the likelihood back up the tree in Felsenstein’s pruning algorithm Felsenstein (1981). These rates obey the w-conditioned reverse of the x-conditioned reverse of the bare mutation operator. From Equation (3), a second application of Bayes rule yields off-diagonal mutation rates

(7) $$\mu_{gg^{\prime }}^{\mathrm{lineage}}(x)=\mu_{gg^{\prime }}\frac{w_{g^{\prime }}(x)}{x_{g^{\prime }}}\frac{x_{g}}{w_{g}(x)}\;\text{\textbackslash{},for}\;g\neq g^{\prime }$$

This apparent forward mutation operator only depends on $\Psi (x)=\log\;\frac{w_{g^{\prime }}(x)}{x_{g^{\prime }}}$. We can thus write the forward mutational operator as a function of Ψ(x). Defining

(8) $$\mu_{gg^{\prime }}^{\left(\Psi (x)\right)}\equiv \mu_{gg^{\prime }}e^{\Psi_{g^{\prime }}(x)-\Psi_{g}(x)}-\delta_{gg^{\prime }}\sum_{h}\mu_{gh}e^{\Psi_{h}(x)-\Psi_{g}(x)}.$$

with $\mu_{gg^{\prime }}^{\mathrm{lineage}}(x)=\mu_{gg^{\prime }}^{\left(\Psi (x)\right)}$. Along lineages, we see the baseline neutral mutation rates become tilted. Transitions toward genotypes with a higher fitness potential ($Psi_{g^{\prime }}>\Psi_{g}$) are exponentially enhanced, whereas transitions toward lower potentials are suppressed. Equation (8) provides the bridge between our backward-time-propagating potential and forward-time phylogenetic inference.

#### Approximate splitting between state and frequency dependence

For the lineage dynamics Equation (8) to decouple from population state, the potential differences $\Psi_{g^{\prime }}(x)-\Psi_{g}(x)$ must be independent of x. At the same time, the normalization constraint $\sum_{g}x_{g}e^{\Psi_{g}(x)}=1$ requires that the individual potentials $\Psi_{g}(x)$ themselves must remain x-dependent. $\Psi_{g}(x)$ must therefore take an additively separable form, composed of a genotype-specific term $\psi_{g}$ that determines the mutational dynamics along the lineage, and a shared log-normalization factor ϕ(x). This shared term cancels upon subtraction in Equation (8), but provides the required x-dependence to enforce the normalization constraint:

(9) $$\Psi_{g}(x)=\psi_{g}-\phi (x)\;\mathrm{where}\;\phi (x)=\log\;\left(\sum_{g}x_{g}e^{\psi_{g}}\right).$$

Substituting Equation (9) into the exact fitness potential Equation (6) and isolating the *g*-dependent terms gives

(10) $$-\partial_{t}\psi_{g}(t)=\sum_{g^{\prime }}\mu_{gg^{\prime }}\left(e^{\psi_{g^{\prime }}-\psi_{g}}-1\right)+f_{g}-\frac{1}{N_{e}}e^{\psi_{g}-\phi (x)}$$

Exact separation is obstructed by the frequency-dependent Ito-drift term $\frac{1}{N_{e}}e^{\psi_{g}-\phi (x)}$.

An allele-independent shift of $\psi_{g}\to \psi_{g}+\xi (x)$ will also, via Equation (9), shift ϕ(x)→ϕ(x)+ξ(x), leaving the full potential $\Psi_{g}(x)=\psi_{g}-\phi (x)$ unchanged. As shown in Appendix: Gauge freedom and frequency-decoupling, this gauge freedom can be used to absorb the frequency-dependence in the Ito-drift term at linear order, yielding the approximate decoupled ODE:

(11) $$-\partial_{t}\psi_{g}(t)=\sum_{g^{\prime }}\mu_{gg^{\prime }}\left(e^{\psi_{g^{\prime }}-\psi_{g}}-1\right)+f_{g}-\frac{1}{N_{e}}\psi_{g}.$$

Equation (11) holds up to order $O(\frac{1}{N_{e}}(\psi_{g}-\phi (x){)}^{2})$, and is thus valid in the weak-selection limit ($\sigma =2N_{e}\Delta f\ll 1$), or for selection acting over short timescales ($\frac{2\Delta f(T-t{)}^{2}}{N_{e}}\ll 1$).

The isolated ψ fully specifies the forward mutation operator,

(12) $$\mu_{gg^{\prime }}^{\psi }=\mu_{gg^{\prime }}e^{\psi_{g^{\prime }}-\psi_{g}}-\delta_{gg^{\prime }}\sum_{h}\mu_{gh}e^{\psi_{h}-\psi_{g}}$$

This establishes that in the weak-selection limit, Equation (11) and Equation (12) define the lineage dynamics without reference to the population state x.

### Connection to optimal control

Our derivation of the frequency-independent fitness potential uncovers a deep duality: where the weak selection limit is valid, the equations governing ancestral lineages are formally identical to those of optimal control theory. (See Control theoretic view of evolving populations for details) In this duality, biological alleles map to agent states, and our fitness potential maps to the control theorist’s “value function”—a measure of maximum future reward. This is not accidental, we defined w and *Ψ*, guided by this duality, specifically to recover and exploit Equation (11), which maps to the Hamilton-Jacobi-Bellman (HJB) equation, the fundamental equation of control theory.

Beyond furnishing a tractable ODE for lineage dynamics, the duality between optimal control and Darwinian evolution provides a mathematical basis for common biological intuitions. Pathogens are commonly personified as clever adversaries-cancer deleting TP53, or bacteria upregulating *β*-lactamase. By interpreting Equation (12) as a mutational control policy, this apparent “evolutionary cleverness” maps directly onto the concept of optimal play. Because we only observe lineages that succeeded in surviving to the present, their historical mutational trajectories mathematically mimic an optimally controlled path-appearing to anticipate and steer around the challenges posed by environmental shifts or mitigation efforts. This anticipation does not arise from biological foresight; it is the strict mathematical inevitability of conditioning the forward dynamics on survival. At the same time, Equation (11) establishes that the capacity for this retrospective anticipation is limited, becoming exponentially suppressed beyond the population’s coalescence horizon, $N_{e}$.

## Results

### Ancestral stationary distribution of a biallelic system

Let us compare our theory to the exact steady-state biallelic distribution to determine its range of validity in a tractable case. For simplicity, $\mu_{12}=\mu_{21}$ and $f_{1}=0$, $f_{2}=f$.

#### The weak-selection approximation compared to selective sweeps

The stationary condition for the fitness potential is given by setting the derivative of Equation (11) equal to zero yields an implicit equation for the potential difference $\Delta \psi =\psi_{2}-\psi_{1}$:

(13) $$\Delta \psi =\text{arcsinh}\;\left(\frac{\sigma }{2\theta }-\frac{\Delta \psi }{\theta }\right)$$

where $\sigma =2N_{e}f$ and $\theta =2N_{e}\mu$. The behavior of Δψ has three distinct regimes depending on the diversity *θ* and the selection ratio f/μ:

(14) $$\Delta \psi =\left\{ \begin{array}{cc} N_{e}f\; & \text{( nearly-neutrally: }\;\sigma <1,\theta <1)\\ \log\;(f/\mu )\; & \text{( purifying-selection: }\;\sigma /\theta >1,\sigma >1)\\ f/(2\mu )\; & \text{( mutation-mixed: }\;\sigma /\theta <1,\theta >1) \end{array}\right.$$

**Fig. 2.**
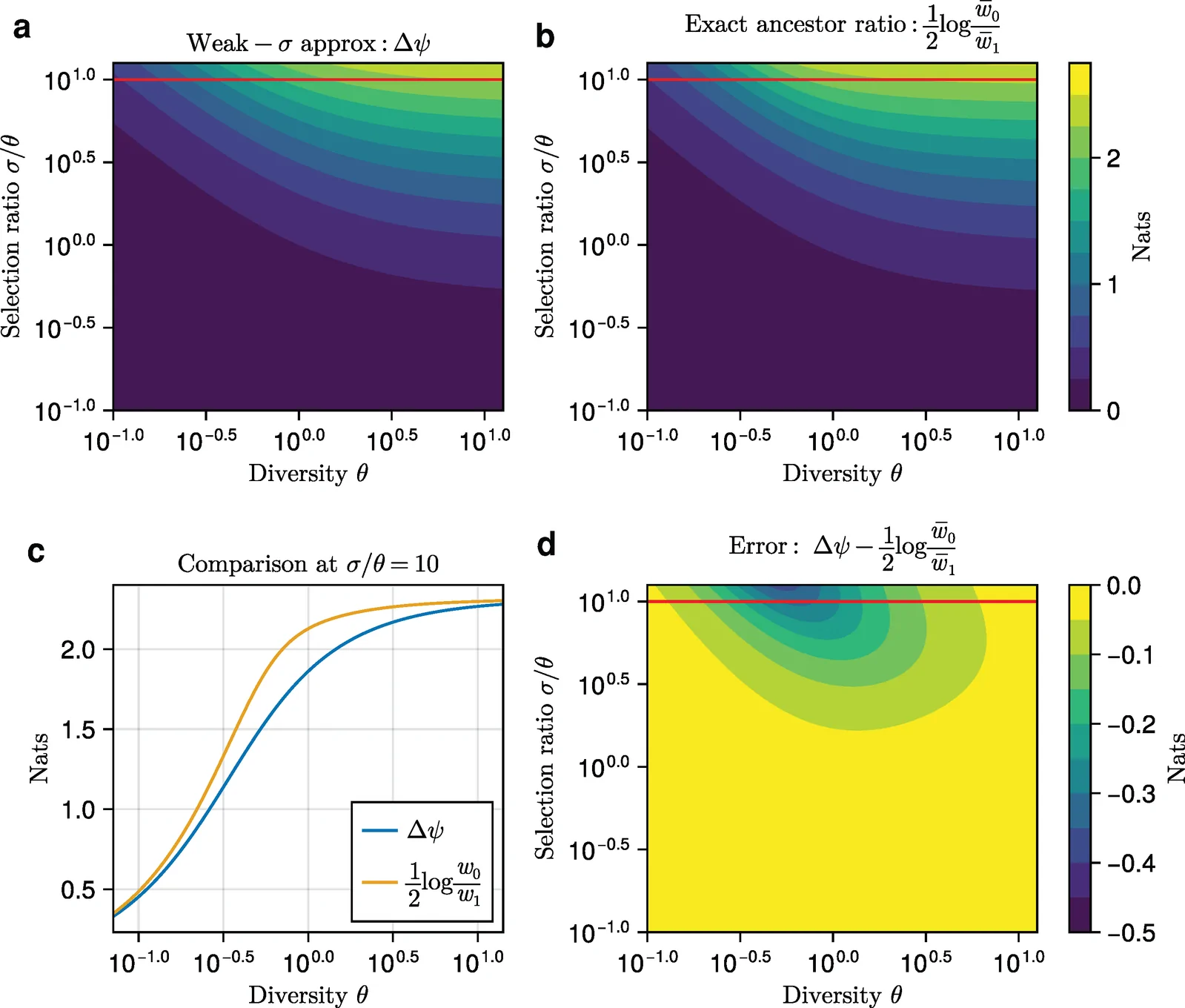
A comparison between the fitness potential estimated from weak selection approximation (Δψ shown in a)) and the exact ancestor ratio b), $\frac{1}{2}\log\;\frac{{\bar{w}}_{0}}{{\bar{w}}_{1}}$, where ${\bar{w}}_{0}$ and ${\bar{w}}_{1}$ have had their frequency dependence averaged over the equilibrium distribution. c) A direct comparison between the two measures as a function of the diversity at a fixed selection ratio of σ/θ=10. d) The comparison in the context of the overall error (line shows the slice in panel c) over the same parameter values as panel a and b, with the greatest error for intermediate values of *θ* and large selection, corresponding to the regime of strongly-correlated clonal interference.

These regimes are sketched in Fig. 2.

#### Comparison to exact results

We now compare this approximation to exact results. We can consider how the weak selection theory compares to stationary ensemble averages,

(15) $${\bar{w}}_{g}=\underset{x∼p_{\mathrm{stationary}}}{E}\;w_{g}(x)$$

In the biallelic case, the stationary distribution for the population is given by the Wright equilibrium equation:

(16) $$p_{\mathrm{stationary}}(x_{2})=\frac{e^{\sigma x_{2}}(1-x_{2}{)}^{\theta -1}(x_{2}{)}^{\theta -1}}{Z(\sigma ,\theta )}$$

##### Rare Mutation Limit (θ≪1)

In this limit, the population toggles between two completely homogenous states. Transitions are governed by the Kimura fixation probability, leading to forward and backward transition rates of $\mu_{12}^{\mathrm{an}}=\mu_{12}\frac{\sigma }{1-e^{\sigma }}$ and $\mu_{21}^{\mathrm{an}}=\mu_{21}\frac{\sigma }{e^{-\sigma }-1}$. The weak-selection approximation recovers the exact ratio of these rates, and consequently the stationary distribution, for arbitrary *σ*:

(17) $$\frac{\mu_{21}^{\mathrm{an}}}{\mu_{12}^{\mathrm{an}}}\;=\;\frac{\mu_{21}^{\psi }}{\mu_{12}^{\psi }}=e^{\sigma }$$

This equivalence holds outside the weak-selection regime, despite the violation of the linearizing assumptions used to derive the biased mutational operator $\mu^{\psi }$. If the mutation operators obey detailed balance, the weak-selection approximation recovers of the correct ancestral distribution for rare mutations with arbitrarily strong selection.

##### Clonal interference regime (Intermediate *θ*)

To study regimes where the weak-selection result leads to error, we must calculate the exact stationary ancestral distribution. While complex analytical results exist Fearnhead (2002), a simpler approach is to solve our equation for $w_{g}(x)$ (Equation (4)) numerically using the Galerkin method with the necessary boundary conditions $w_{1}(x_{1}=1)=1$ and $w_{2}(x_{2}=1)=1$ (i.e. the ancestor must be the type of the fixed allele). Comparing these exact numerical results to our approximation (Fig. 2) confirms that our theory is accurate at the extreme limits of all three regimes. The greatest errors occur, as expected, at intermediate values of *θ* combined with strong selection, where clonal interference is most significant.

### Anticipatory lineage dynamics

**Fig. A1.**
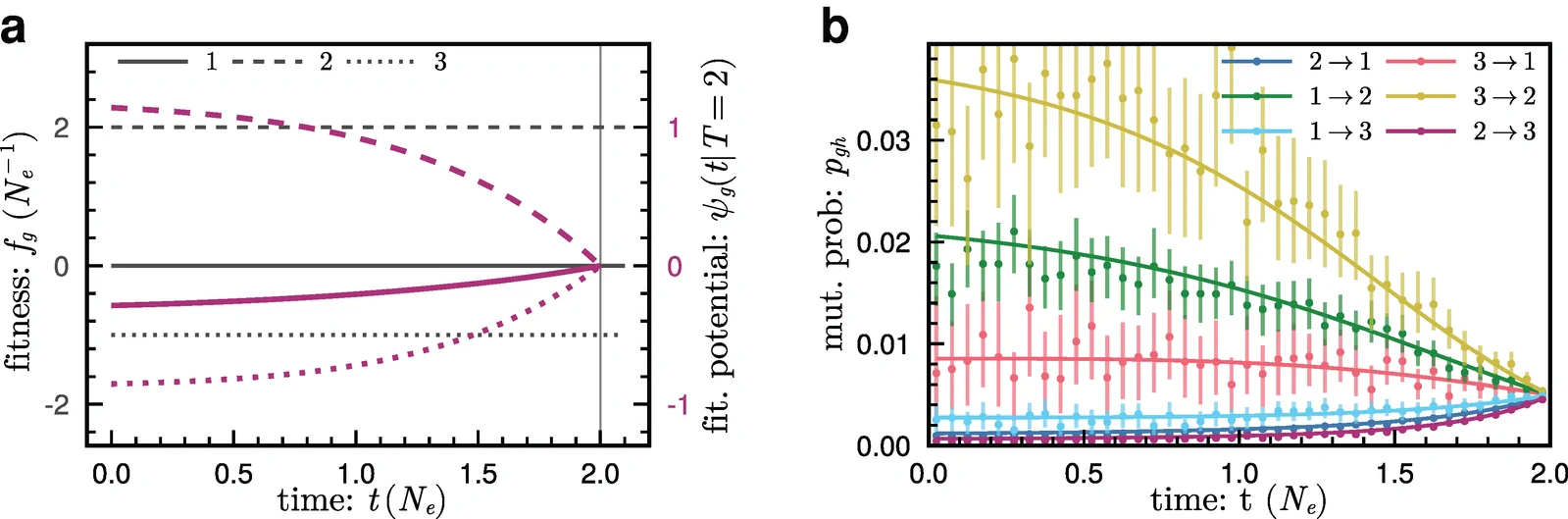
Constant fitness. Panel a) A triallelic system with constant fitness differences has a fitness potential which exponentially saturates at the scaled selection coefficients σ . Panel b) The mutations t along an ancestral lineage obey a biased mutational operator $\mu^{\psi }$ governed by *ψ*. Near the sample time $2N_{e}$, the mutations approach their neutral values, tracing them backward in time they approach their equilibrium values, governed by $\mu e^{\sigma }$.

**Fig. A2.**
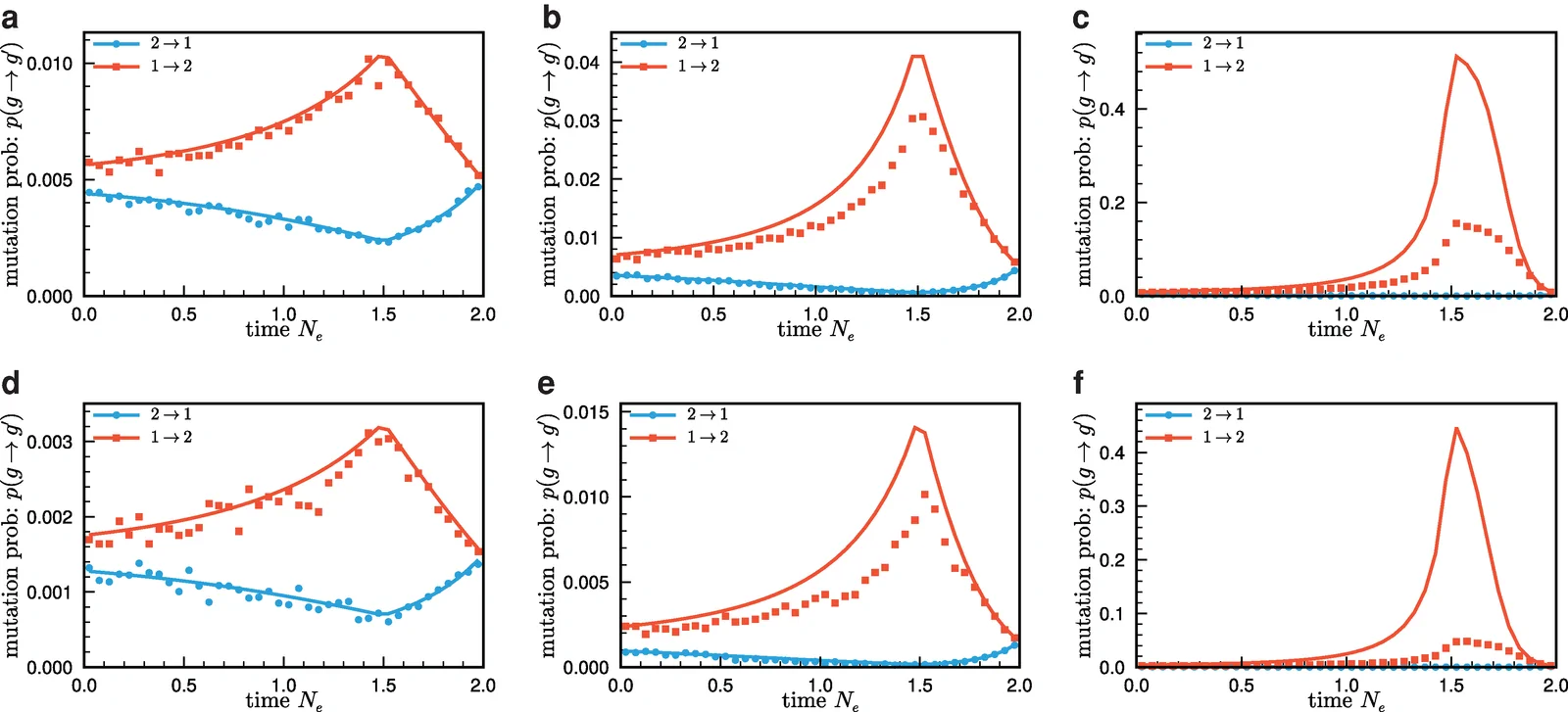
Breakdown of theory with strong positive selection. Shown are the results for the biallelic pulsed fitness model (between 1.5 and 2.0 $N_{e}$) sampled at $2.0N_{e}$, for panel a–c θ=0.03, and panel d–f θ=0.01 with increasingly strong selection from left to right . When mutation rates are low and selection is stronger, we see that theory predicts more mutational biasing than actually occurs. The dependence on the mutation rate in the prediction error (panel c vs panel f) is a result of discrete individual effects not captured by the Kimura diffusion operator which neglects the effects of mutational shot-noise.

We now apply our formalism to dynamic, non-equilibrium scenarios, focusing on the biologically relevant case of time-varying fitness with constant mutation rates. We numerically solve the fitness potential ODE and use it to compute the mutation propagator. We then compare these theoretical predictions to mutation rates observed along lineages in stochastic simulations. We deliberately choose parameter regimes where our weak-selection approximation begins to break down, demonstrating that even in these challenging cases, theory captures the essential qualitative features of the ancestral process. Further models, a triallelic system (Fig. A1), and a study of the breakdown of the weak-selection approximations (Fig. A2), are shown in App. Additional models.

#### Fitness pulse

We consider a brief selection event between 1.6 and 1.8 $N_{e}$, which is negative for allele 1 and positive for allele 2. We might imagine this as a brief intervention, such as antibiotic therapy acting on existing gut microflora or an anticancer agent briefly applied to a tumor. The mutational process is symmetric $\mu_{12}=\mu_{21}=\mu =0.1/N_{e}$. The fitness function is

(18) $$f_{g}=a_{g}(\Theta (t-t_{0})-\Theta (t-t_{1}))\;\text{with}\;\left\{ \begin{array}{c} a_{1}=-5\times N_{e}^{-1}\\ a_{2}=5\times N_{e}^{-1} \end{array}\right.$$

**Fig. 3.**
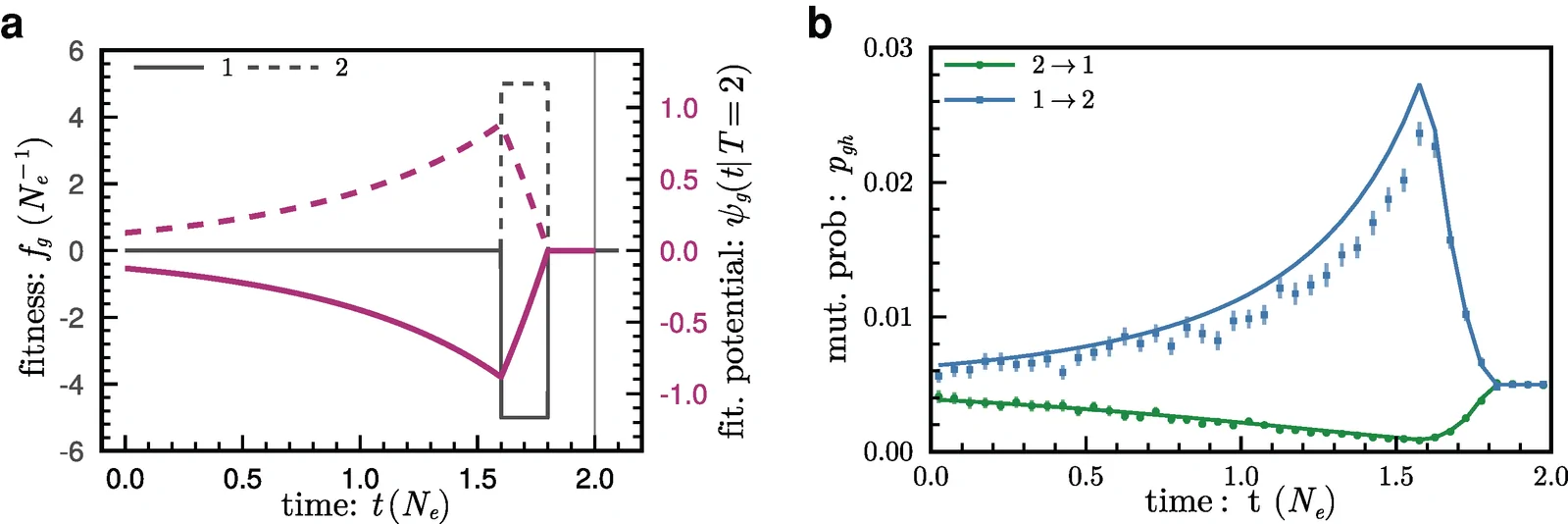
Brief strong selection. a) We consider a brief selection event between 1.6 and 1.8 $N_{e}$ on a biallelic population with θ=0.1, balanced mutation rates (gray lines). Allele 1 experiences negative selection (solid lines) and allele 2 experiences positive selection (dashed lines). Tracing a lineage sampled at time $2N_{e}$ backward in time, it experiences a selection potential given by the purple lines. As a result the mutational operator acting along the lineage, $\mu^{\psi }$ governed by *ψ*, is biased before the selection pulse. The mutational process b) along a lineage is anticipatory in that it occurs before the selection event. The mutation probability, observable in the number of transitions along the lineage over discrete intervals of $N_{e}/20$ recapitulates the predictions of theory. Since θ=0.1, this predicts a neutral mutation rate of $p_{gg^{\prime }}=5\times 10^{-3}$ observed after the selection pulse is over. During and prior to the selection pulse, the fitness potential becomes higher for allele 2 than for allele 1 and mutations into the positively selected allele (2) are enhanced, and the negative allele (1) are reduced.

The intensity of the fitness pulse is given in units of $N_{e}$ and if applied continuously would be associated with an enormous selection coefficient σ=20. Tracing a lineage backward in time sampled at time $2N_{e}$, after the pulse is over, it experiences a selection potential given by the blue lines in Fig. 3. We see that the mutational operator acting along the lineage, $\mu^{\psi }$ governed by *ψ*, becomes increasingly biased prior to the selection pulse.

The mutation rates along a lineage appear to be subject to an optimal controller trying to drive the lineage into the positively selected state 2. This control slowly turns on prior to the fitness pulse, and turns back off as we approach the end of the pulse. In this sense, the process as anticipatory, though this anticipation is purely the result of *post-hoc* conditioning a lineage on having surviving offspring at the sampling time *T* (in Fig. 3 $T=2N_{e}$). Looking at the distribution of mutations along a lineage as shown in Fig. 3, we confirm that the mutational biases decay as the fitness potential decays exponentially at rate $1/N_{e}$, validating that the coalescent time-scale $N_{e}$ sets the discounting factor in the dual control problem.

#### Seasonal adaptation in viral infections

Suppose we have two phenotypes of an influenza strain, one of which is summer-adapted, and one of which is winter-adapted. We can write the fitness function as

(19) $$f_{g}=a_{g}\sin\;\left(\frac{2\pi t}{T_{\mathrm{period}}}\right)\;\text{with}\;\left\{ \begin{array}{c} a_{1}=-2\times N_{e}^{-1}\\ a_{2}=-2\times N_{e}^{-1} \end{array}\right.$$

**Fig. 4.**
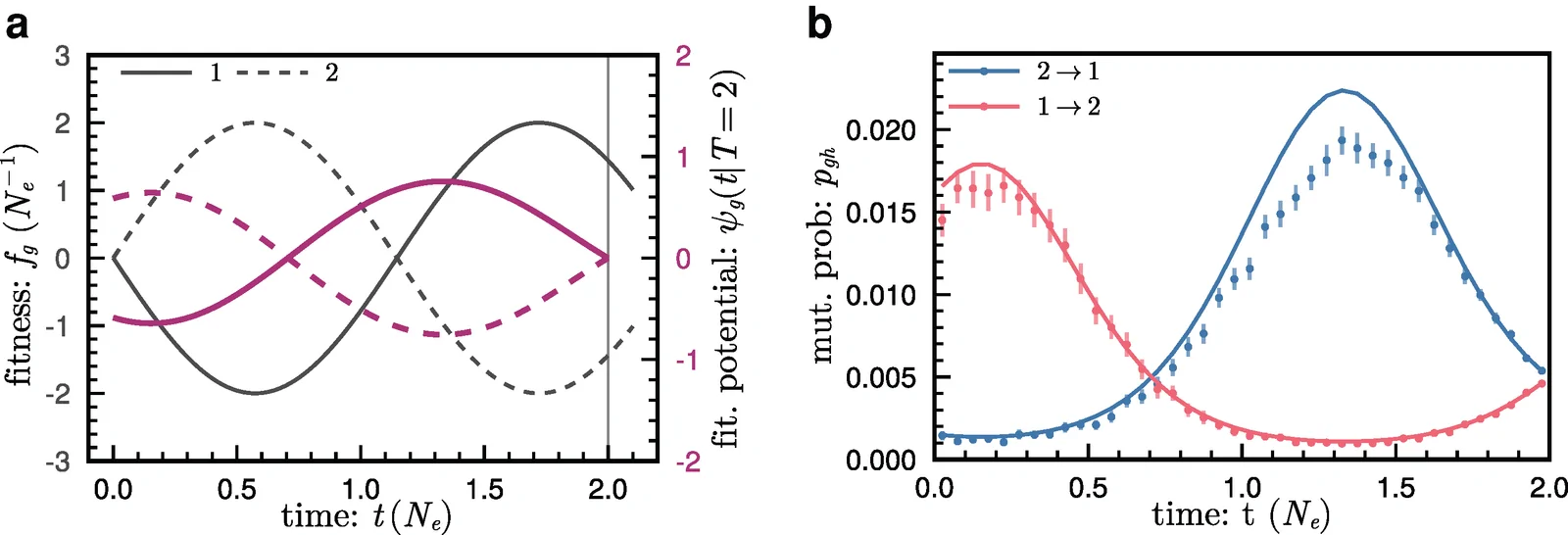
Sinusoidally varying selection. We simulate the effect of sinusoidal selection (panel a, gray lines) on a biallelic population with θ=0.1, and balanced mutation rates. Tracing a lineage sampled at T=2.0 backward in time, it experiences a selection potential given by the purple lines. b) As a result the mutational operator acting along the lineage, $\mu^{\psi }$ governed by *ψ*, is biased before the selection pulse. The mutational process is anticipatory in that it occurs before the selection event, leading to a negative phase shift relative to the selection itself.

In Fig. 4, $T_{\mathrm{period}}=2.3N_{e}$. Considering the living population, obeying the diffusion equation with a time varying flux, observed phenotypes in the living population are lagging the fitness effects since the living phenotype distribution is forward integral of the Kimura equation. Thus the summer-adapted strains are, broadly speaking, most dominant in the fall after the cumulative effect of the entire summer of fitness pressure.

The situation is quite different for the ancestral distribution. Because the fitness potential is the backward-time integral of the fitness, the mutational bias in the ancestors is strongest in the *preceding* season: mutations establishing the summer-adapted strain are most over represented in the spring, and for the winter-adapted strains in the fall. From the optimality perspective, the ancestral distribution shifts to summer-adaptation early to maximize the benefit of a summertime fitness advantage Fig. 4.

## Maximum likelihood inference on reconstructed trees

Suppose that we observe a genotype $G_{1},G_{2}\ldots$ in a populations at several sample times $T_{1},T_{2}\ldots$ and we can reconstruct the tree topology T for the gene from linked neutral variation with minimal uncertainty. First we will calculate the exact likelihood and describe the path to its complete evaluation. Then we will show how the weak result allows the calculation of the likelihood in a more tractable form.

### Exact likelihood particle methods

We construct the exact likelihood given a particular frequency history *X*, a path through frequency space governed by the Fisher-Kimura stochastic generator *L*.

(20) $$P(G,T\;|\;\Theta )=\underset{X\;|\;L(\Theta )}{E}\;P(G\;|\;X,\Theta )\times P(T\;|\;G,X,\Theta )$$

Here, $P(G\;|\;X,\Theta )=\prod_{i}x_{G_{i}}(T_{i})$ represents multinomial sampling at each time point. The difficult term is the expectation $\underset{X\;|\;L(\Theta )}{E}$, which requires integrating over a vast, high-dimensional space of possible frequency paths. Evaluating this integral is computationally intractable for realistic datasets, often requiring expensive particle filter methods (see Exact inference using the lineage frequencies and marginalization over paths for a detailed formulation).

### Weak-selection likelihood

In the weak-selection limit, we can leverage the frequency independence to dramatically reduce the effort required to evaluate the likelihood of genotypic data. Instead of using Monte Carlo sampling to estimate the integral over *X*, we can analytically marginalize out these hidden paths.

We begin by separating the probability of the tree topology and the probability of the data, given the tree.

(21) P(G,T|Θ)=P(T|Θ)×P(G|T,Θ)

The tree likelihood is (in the same weak-limit) well approximated by the neutral coalescent, with lineage independent hazard $\frac{1}{N_{e}}$. For coalescent times $t_{c}$,

(22) logP(T|Θ)=logP(T|Ne)=∫t(|A(t)|2)Ne(t)dt−∑clogNe(tc)

Where A(t) is the set of lineages alive at time *t*, and (|A(t)|2) counts the number of interacting pairs.

To evaluate the data likelihood P(G|T,Θ), we must account for the interactions between lineages due to coalescence hazard. Derived in Inference-methods and tree likelihood, coalescence induces an interaction term in the fitness potential $\psi^{i}$ of lineage *i*, This term combines with the single-lineage dynamics to yield lineage-coupled ODEs:

(23) $$-\partial_{t}\psi_{g}^{i}=\sum_{g^{\prime }}\mu_{gg^{\prime }}\left(e^{\psi_{g^{\prime }}^{i}-\psi_{g}^{i}}-1\right)+f_{g}-\frac{1}{N_{e}}\sum_{k\in A(t)}\psi_{g}^{k}$$

At the end of a lineage $T_{i}$, where children C(i) coalescen into a parent *i*, we have the boundary condition

(24) $$\psi_{g}^{i}=\left\{ \begin{array}{cc} \sum_{\;j\in C(i)}\psi_{g}^{\;j}\; & \text{edge}\;i\;\text{with children}\;C(i)\\ 0 & \text{edge}\;i\;\text{terminating in an observation} \end{array}\right..$$

The data likelihood can be computed efficiently using a standard recursive pruning algorithm. The fitness potential defines a time-dependent, biased mutational operator $\mu^{\psi^{i}}(t)$ Equation (12) along each branch *i*. These $\mu^{\psi^{i}}(t)$ are the generator for the Kolmogorov equation, integrated backward from the tip:

(25) $$-\partial_{t}p_{gg^{\prime }}^{i}(t)=\sum_{k}\mu_{gk}^{\psi^{i}}(t)p_{kg^{\prime }}^{i}(t)$$

with the boundary condition at the tip, $p_{gg^{\prime }}^{i}(T_{i})=\delta_{gg^{\prime }}$. The likelihood is then found recursively from the tips to the root:

(26) $$L_{g}^{i}=\left\{ \begin{array}{cc} \sum_{g^{\prime }}p_{gg^{\prime }}^{i}\prod_{\;j\in C(i)}L_{g^{\prime }}^{j}\; & \text{\textbackslash{},for edge}\;i\;\text{with children}\;C(i)\\ p_{gG_{i}}^{i} & \text{\textbackslash{},for edge}\;i\;\text{terminating in}\;G_{i} \end{array}\right.$$

Then the total probability of th eobservations given the tree is

(27) $$P(G\;|\;T,\Theta )=\sum_{g}\pi_{g}L_{g}^{\mathrm{root}}$$

where π is a chosen prior (see Observation likelihood from biased-mutational operator) over the common-ancestor state.

### Inference of seasonal parameters

**Fig. 5.**
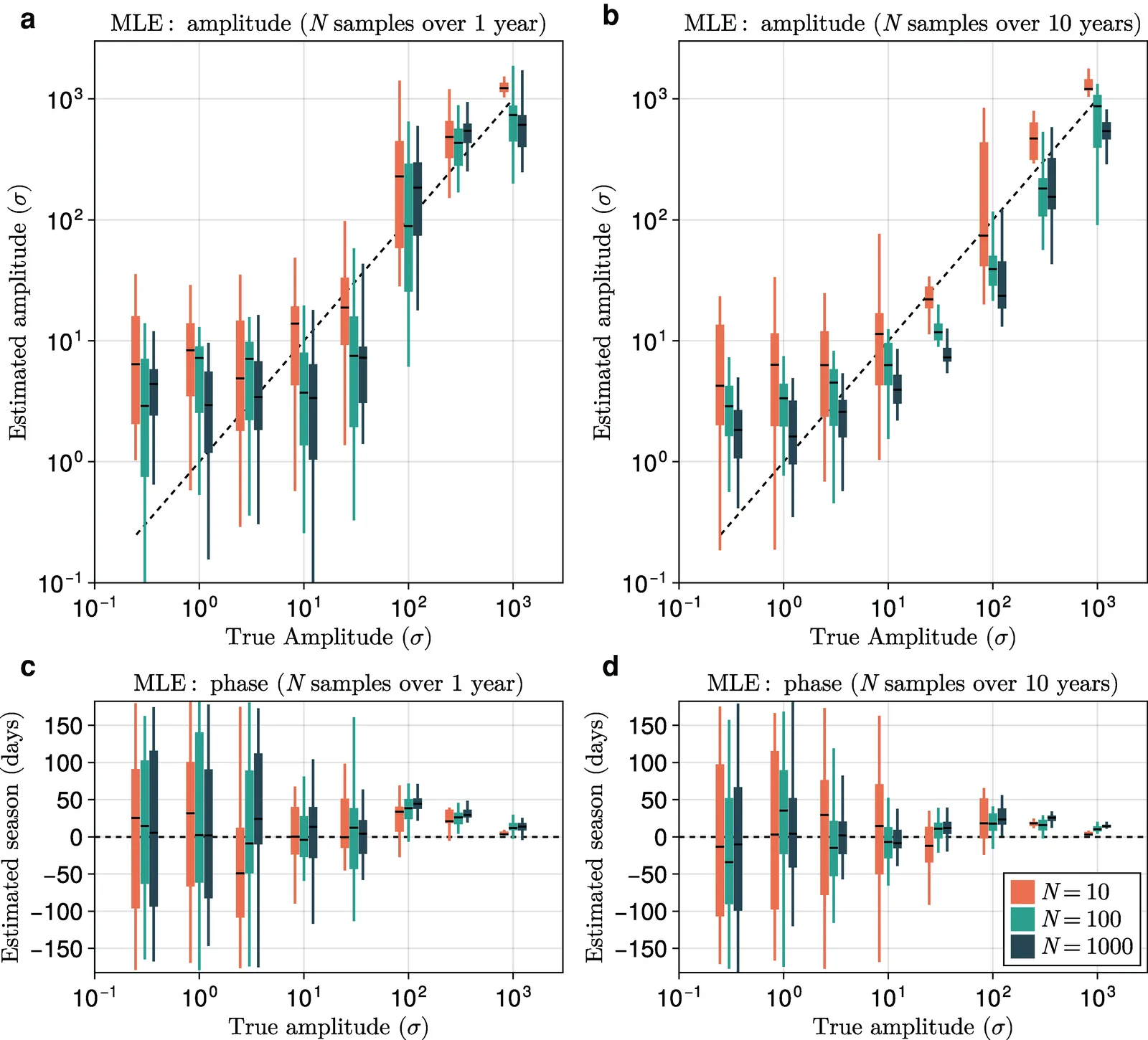
Inferring amplitude and phase of seasonal selection. We plot the distribution of maximum likelihood estimators (MLEs) for the amplitude and phase of the seasonal selection model where true parameters are $a=2/N_{e}$, and ϕ=0. The population size is $10^{3}$, and the diversity parameter is θ=0.1 for 10, 100 and 1,000 samples. In panels a and c (b and d) the observations are spread over a single year (10 years). Overfitting biases the inferred amplitude high (especially with only 10 samples N=10), and fit a random phase (c and d). At long obersrvation time and strong-selection the phase inference undergoes a transition and collapses near the true value. However, distortions from the weak-selection approximation generates a small phase bias and leads us to underinfer selection even with N=1000.

We can infer the parameters of the seasonal selection model on trees generated from Monte Carlo population simulations. We find that we can collapse the phase parameter and infer the amplitude of the selection even for moderately-weak selection pressure Fig. 5, even though the time-averaged fitness difference between the alleles is zero and the mutation rate is low (θ=0.1). The bias due to our weak-selection theory distorts some of the absolute values of the inference parameters from their true values in the simulation.

Comparing the 1 yr observational span and the 10 yr span, increasing the sampling timeframe is comparable to increasing number of observations in terms of decreasing the maximum-likelihood estimator (MLE) variance and resistance to overfitting. This is because spacing observations out further gives us more effectively independent seasons in which to see the one allele takeover the other.

## Discussion

### Conceptual significance of control duality

The derivation of the Hamilton-Jacobi-Bellman (HJB) equation from the lineage distribution relies on viewing Darwinian evolution as a naturally occuring particle filter (see Appendix Control theoretic view of evolving populations). In a particle filter, the target value is read out from the retrospective enrichment of successful lineage states $\log\;(w_{g}/x_{g})$ Del Moral (2011). This target defines the fitness potential. This algorithmic view justifies the decoupling of the lineage dynamics from the population state x: If Darwinian evolution is a natural particle filter, the targeted dynamics (the optimal control) should be asymptotically independent of the population state. In particle-filter theory, the population state is merely an algorithmic hyperparameter affecting sample efficiency. The derivation of Equation (12) realizes this property, demonstrating that the dynamics of surviving lineages lose their x-dependence in the weak-selection limit.

Identifying the target of the particle filter as an HJB equation yields a dictionary between evolutionary forces and the elements of stochastic control. Fitness *f* acts as the reward function, the forward mutation rate *μ* as the uncontrolled dynamics, and the coalescent timescale $N_{e}$ as the discount factor governing the integration time of the reward. Conditioning descent on survival to time *T* defines a forward dynamics generated by the biased mutational operator $\mu^{\Psi }$, which corresponds to the generator for the optimal policy. Finally, the fitness potential *ψ* is the local generating function for this optimal solution-the value function.

Equating lineage paths with optimally controlled trajectories has two immediate consequences. First, it establishes that surviving lineages behave as rational agents optimizing a tradeoff between fitness rewards and the entropic cost of mutational steering. This perspective clarifies the counterintuitive backward-propagating effect of survivorship bias. Second, this equivalence provides a constructive method for building the lineage transition rates. Rather than attempting to integrate the x-dependent Kimura diffusion directly, the Bellman formalism Kirk (2004) dictates a specific solution sequence: One first solves the HJB equation for the value function (*ψ*), and then uses this potential to explicitly construct the generator for optimal control ($\mu^{\psi }$). We recover this exact control-theoretic sequence from within population genetics, (Equations (11) and (12)). This allows evolutionary inference that avoids the computationally prohibitive high-dimensional integration of direct methods.

### Relation to existing theories

#### Current approaches to phylogentic inference

Inference strategies generally fall into two categories: “tree methods” and “trajectory methods.” Despite their respective limitations, both approaches possess technical merits that make them appropriate for state-of-the-art inference. Shimagaki et al. (2025), Bouckaert et al. (2014) and Lewinsohn et al. (2023).

Tree methods typically employ a birth–death-sampling model Stadler et al. (2013) and Kühnert et al. (2016). The central trick of these models (Maddison et al. 2007) is that if we keep track of the probability of an individual *not* being sampled at any future time, the birth–death model P(G,T|Θ(X)) becomes directly integrable via Felsenstein pruning. However, to maintain this tractability, standard implementations typically treat lineages as conditionally independent entities governed by piecewise-constant rates (e.g. skyline models). This decoupling introduces two fundamental limitations. First is over-parameterization: by ignoring the mechanistic constraint that couples growth rates to the population state ($\beta_{g}-\delta_{g}=f_{g}-\bar{\;f}(x)$), these models require an excess of parameters to describe dynamics that are physically generated by a small number of selection coefficients acting on a changing background. Second, and importantly for endemic populations, is the noise structure. By treating lineages as independent branching processes, these methods implicitly assume the variance structure of an infinite population (where variance scales with birth rate). They therefore fail to capture the information decay inherent to finite populations (where variance scales with drift, $∼1/N_{e}$), potentially overestimating the confidence of inference in the deep past (see The birth–death noise structure).

Trajectory methods, in contrast, study the frequency paths $\underset{X\;|\;L(\Theta )}{E}\;P(G\;|\;X,\Theta )$ directly. Here, fitness effects are observed via their impact on frequency changes in accordance with the replicator dynamics, $\partial_{t}\log\;x_{g}=f_{g}-\sum_{g^{\prime }}x_{g^{\prime }}f_{g^{\prime }}$ Seifert et al. (2015), Sohail et al. (2022), Shimagaki et al. (2025), Shekhar et al. (2013), Ferguson et al. (2013), Louie et al. (2018) and LaMont et al. (2022). For isolated alleles of interest, the associated Kimura propagator can be evaluated directly via spectral expansions or efficient MCMC sampling Schraiber et al. (2016), Jenkins and Spanò (2017) and Paris et al. (2019). However, applying this method to the space of linked alleles renders the full population propagator intractably high-dimensional. Assuming linkage equilibrium restores tractability but discards the covariation required to distinguish true selection from genetic draft. One alternative is to approximate the joint continuous dynamics using a multivariate Gaussian likelihood Illingworth et al. (2020) and Buffalo and Coop (2020), but this requires the inversion of high-dimensional, ill-conditioned empirical covariance matrices.

Our method bridges this divide: it uses the full resolving power of the tree structure (like birth–death models) but accounts for the lineage-population coupling and finite-population drift (like replicator dynamics) via the fitness potential. Via Equation (4), the correlation between the lineage state and the population state is explicitly handled through the backward Kolmogorov evolution $L_{x}$, giving us the correct noise-structure.

An advantage of the fitness potential is its scalability to high-dimensional genotype spaces. In standard multi-type birth–death models, inference on sequence data requires tracking the joint distribution of all sites to calculate rates, a task that scales exponentially with sequence length ($G^{L}$). Consequently, these methods must coarse-grain sequences into arbitrary “clades.” In contrast, our fitness potential factorizes under an additive fitness model. If we assume additive fitness (independent sites), the total potential decomposes into a sum of site potentials:

(28) $$\psi_{\mathrm{total}}(g)=\sum_{k=1}^{L}\psi_{k}(g_{k})$$

Consider a mutation event that occurs only at site *j*, changing genotype $g\to g^{\prime }$. Since $\psi_{g^{\prime }}$ and $\psi_{g}$ are identical at all sites except *j*, the difference in potential—which drives the transition probability—depends only on the local contribution:

(29) $$\Delta \psi_{\mathrm{total}}=\left(\psi_{j}^{\prime }+\sum_{k\neq j}\psi_{k}\right)-\left(\psi_{j}+\sum_{k\neq j}\psi_{k}\right)=\psi_{j}^{\prime }-\psi_{j}$$

The probability of a transition at site *j* therefore depends only on the fitness potential at site *j*, with the complex background interactions absorbed into the shared normalization. This decomposition allows for site-specific fitness inference at scale.

#### The landscape of reproductive value

Our fitness potential Ψ(x,t) generalizes Fisher’s concept of reproductive value to dynamic, non-equilibrium populations Fisher (1999). While tractable transition rates require the weak-selection limit, the definition of the fitness potential applies to any Markovian population with state x and generator $L_{x}$. In the limit of constant fitness and infinite time horizons, *Ψ* converges to the logarithm of the dominant left eigenvector of the evolutionary generator—exactly recovering Fisher’s classical definition. However, for time-varying selection (such as seasonal adaptation) or finite observation windows, *Ψ* captures the instantaneous reproductive value: a time-dependent field that guides lineages not toward a static optimum, but toward survival at the specific sampling time *T*.

The standard fitness landscape f(x) describes the instantaneous forces (birth and death rates) acting on the population. In contrast, the fitness potential Ψ(x) describes the integrated value of a state. In physics terms, if fitness *f* acts as a local force or gradient driving frequency changes, then *Ψ* acts as the potential energy surface. By transforming the problem from the local landscape (*f*) to the global potential landscape (*Ψ*), we move from describing the causes of evolution (selection) to describing the local maximization that takes place along the ancestral lineages.

Because *Ψ* integrates parameters such as *f*, *μ*, and $N_{e}$ into a single scalar field, shifts in any structural parameter correspond to a change ΔΨ. *Ψ* thus defines a general effective scaled selection coefficient $2\Delta \Psi (x,t,T)\approx \sigma_{\mathrm{eff}}(x,t,T)$, establishing a common metric for disparate evolutionary forces. For example, consider the deletion of TP53 during tumor progression. The deletion imposes an immediate replication penalty (Δf<0) but increases the variance of *μ*. The control framework specifies that a net positive $\sigma_{\mathrm{eff}}$ emerges exactly when the altered *μ* sufficiently reduces the entropic cost of reaching future high-fitness genotypes within a specified timescale T−t. The potential *Ψ* thus calculates the trade-off between instantaneous replication and long-term access to adaptive trajectories, providing a retrospective dual to the “survival of the flattest” phenomenon in quasispecies theory Wilke et al. (2001), Sardanyés et al. (2008) and Wilke (2005) and the selection for evolvability in modifier theory Hansen (2006), Wagner and Altenberg (1996) and Lynch (2010).

#### Relationship to quasispecies theory

In the infinite population limit ($N_{e}\to \infty$), the exponentiated value function $z_{g}=e^{\psi_{g}}$ follows a linear operator equivalent to the quasispecies equation, $\partial_{t}z+(\mu +f)z=0$. This connection is a consequence of the Feynman-Kac lemma, which relates the stochastic Bellman equation to a linear path integral. The reverse mapping, a logarithmic transformation of the quasispecies density, yields a forward-time Hamilton-Jacobi equation mathematically close to Equation (11) Ancliff and Park (2011), Domingo et al. (2012), Baake and Alberti (2021) and Rouzine et al. (2003). Through this logarithmic transformation, it is possible to derive equilibrium results and perturbation theories for complex fitness landscapes Diekmann et al. (2005), Saakian (2007), Saakian et al. (2019) and Saakian and Hu (2016).

Despite sharing mathematical resemblance, the physical interpretation is distinct. Quasispecies theory describes the forward evolution of the population log-density $\log\;x_{g}$ Biebricher and Eigen (2006), Eigen et al. (1988) and Wilke (2005) in the deterministic infinite population limit ($N_{e}\to \infty$). In contrast, the fitness potential $\psi_{g}$ evolves backward from a future boundary condition (survival at *T*). Furhtermore, the lineage control incorporates finite population effects via the discount rate $\gamma =N_{e}^{-1}$, introducing a nonlinearity in the control cost absent in the deterministic quasispecies limit.

#### Relationship to forward-time population frameworks

We compare the lineage path integral to the population path integrals of non-equilibrium thermodynamics Mustonen and Lässig (2008, 2010). Those formulations assign statistical weights to the continuous histories of the entire population frequency distribution x(t). The framework derived here instead characterizes the distribution of discrete genotypic paths along the ancestral line g(t).

Beyond natural thermodynamic drift, the forward-time population dynamics have also been studied as the target of external control, considering how to bring an eco-evolutionary system to a preferred state at minimal cost Nourmohammad and Eksin (2021) and Lässig et al. (2023). The lineage dual control developed here contrasts with both approaches. It does not describe an external intervention, nor does it model the forward-time drift of the population state. It emerges internally to Darwinian evolution, as a mathematical description of the effect of conditioning on lineage survival to the sample time *T*.

### Limitations

#### Dependence on tree data

The phylogenetic trees used as input are not directly observable but must be reconstructed from sequence data. While tree inference is computationally challenging, significant progress in maximum-likelihood and rapid heuristic methods has made accurate reconstruction feasible even for massive datasets Sagulenko et al. (2018), Van de Peer and Salemi (2009), Kumar et al. (2004), Feng et al. (2006), Flouri et al. (2018) and Price et al. (2010). In this work, we condition on a fixed tree topology, an approach appropriate for the “big data” regime where the phylogenetic signal is strong and topological uncertainty is subordinate to demographic stochasticity.

However, the framework is not mathematically limited to fixed topologies. The fitness-potential likelihood P(T|Θ) derived here constitutes a rigorous probability density for the tree structure under selection and finite-population drift. It is therefore suitable for integration into Bayesian phylogenetic samplers (e.g. BEAST2, RevBayes Laudanno et al. 2021; Höhna et al. 2019; Bouckaert et al. 2014), serving as a physics-aware drop-in replacement for standard coalescent or birth–death priors. This integration would allow future implementations to marginalize over topological uncertainty while retaining the correct mechanistic handling of lineage competition.

#### The limits of the weak-selection approximation

The full fitness potential Ψ(x,t,T) determines ancestral lineage dynamics via Equation (8). *Ψ* depends on x and general phylogenetic likelihoods require integration over the unobserved population history x(t). Under the separable decomposition $\Psi_{g}\approx \psi_{g}-\phi (x)$ the x-dependence drops out of the dynamical equations. Because this decomposition is derived in the weak-selection limit, establishing its domain of validity is necessary for applications where selection is strong (σ≫1).

Exactness at the null hypothesis (σ=0) ensures that the framework is a valid tool for selection detection, even as quantification of *σ* is approximate. The site-specific likelihood (Equation (28)) from linked neutral components naturally provides a reference distribution for a phylogeny-aware likelihood-ratio test.

For selection quantification, the weak-selection approximation introduces a bias in the inferred selection coefficients $\sigma =2N_{e}\Delta f$ when selection is strong (see Appendix: Convexity and inference bias under strong selection) The approximation has discarded the non-linear component of a drift-induced drag on leaving descendants. For positively selected alleles, the result is that we underestimate the fitness advantage required to drive observed dynamics, and the inferred *σ* is a conservative lower bound for positive selection. Conversely, the approximation is expected to overestimate the magnitude of strong negative selection.

The structural reliability of this approximation is supported by its exactness at temporal extremes. Over short timescales, the variation in the fitness potential remains small, rendering the decomposition locally exact (see the discussion after Equation (11)). On long timescales, the weak limit recovers the exact ancestral stationary distribution for mutation-selection balance in the rare-mutation limit (provided the mutational operator obeys detailed balance; see Ancestral stationary distribution of a biallelic system).

Although the approximation outputs an interpretable lower bounds on *σ* in the presence of strong allele-dependent selection, even weak *frequency-dependent* fitness (e.g. Van Valen 1973; Sinervo and Lively 1996) breaks the separability assumption $\Psi \approx \psi_{g}-\phi (x)$ at leading order (Equation (10)). Intuitively, x-conditioning cannot be avoided if we wish to model x-dependent changes in relative fitness.

## Conclusions

A link between control theory and evolution has previously emerged. Particle filters, Monte Carlo methods that mimic Darwinian evolution Kanazawa et al. (1995) and Del Moral (2011), can be used to solve an optimal control problem Kappen (2005a) and Ruiz and Kappen (2017). We have shown that this link can also be reversed to derive a quantitative statistical theory for the ancestral proccess. Based on this theory, the fitness inference method we present can be applied to time dependent fitness models, out of equilibrium. We can infer both positive and negative selection, and we do not require inferring an underlying genotype frequency x. The method makes direct use of linked neutral variation, which is represented by the temporal embedding of the reconstructed tree. However, detailed frequency dependence reemerges in the strong-selection clonally-interfering limit, making the method only approximate for an important class of problems: strong selection over timescales on order the neutral coalescent times ($N_{e}$).

Selection on inherited traits does not specifically require genetic material, and inherited epigenetic states transmitted between generations, or correlated environmental conditions between parent and child could likewise be treated with a control theoretic framework. Evolution and control are linked at a level more fundamental than genetic inheritance itself.

The lens of optimal control theory provides a fascinating perspective on the apparent emergence of adaptive planning. The apparent “stratgies” emerging from Darwinian adaptation can now be mathematically mapped precisely to optimal strategies in a corresponding game of stochastic control. An evolving pathogen appears to be an opponent playing optimally against human mitigation strategies because this optimal play (within the problem constraints) is a general emergent feature of Darwinian adaptation from the perspective of the common ancestral line.

## Supplementary Material

iyag122_Peer_Review_History

## Data Availability

All data underlying this article were generated computationally. The code and resulting digital artifacts are accessible as described in the Code Availability section.

## Code availability

Code for the fitness potential framework and figure generation is available at: https://github.com/chelate/fitness_potential_paper

## AI Usage Statement

Large Language Models (Gemini 2.5, Gemini 3, and ChatGPT 4.1o) were used during the drafting process to assist with iterative editing, refining the narrative, and checking for clarity for a broad audience. The mathematical derivations, conceptual framework, and code implementation (except where noted) were generated by the author. All AI-suggested text and code was critically reviewed, verified, and revised by the author, who assumes full responsibility for the accuracy and integrity of the work.

## Appendix A: Control theoretic view of evolving populations

To introduce the mathematical duality at the heart of our method consider the code for a population-genetic simulation, popgen.c. The program takes as inputs population size, *N* and noise $\lambda =N/N_{e}$, fitness *f* and mutation rates *μ*, as time varying functions. It then generates a population of size *N* and evolves it forward in time with birth and death rates

( A1) $$\beta_{g}=\frac{\lambda +(f_{g}-\sum_{g}x_{g}f_{g})}{2}\;\delta_{g}=\frac{\lambda -(f_{g}-\sum_{g}x_{g}f_{g})}{2}$$

and mutation $\mu_{gg^{\prime }}$ from time 0 to time *T* evaluated with the Gillespie method, keeping track of the lineage relations of the entire population. The same code can be viewed in two ways:

- A population geneticist sees a Moran-type Wright-Fisher simulation
- A control theorist sees a Monte Carlo algorithm (a particle filter) for solving a continuous-time stochastic control problem.

In the following sections we reverse engineer the parameters of the control problem that popgen seems to be solving from the control-theorist perspective.

### Reverse engineering the control problem

We recall the basic principles of optimal control theory. One seeks policies $u_{h}(g,t)$, which determine our action *h* as a function of state *g* and time, so as to maximize expected rewards. Because of the general Markov property, optimal policy is a local function of the expected optimal reward at each state—the value function ψ(g,t). Therefore, constructing the value function amounts to solving the control problem.

#### Path-integral control

The value function satisfies the Bellman equation, a partial differential equation in continuous space. For (typical) high-dimensional systems the Bellman equation is intractable. However, in the remarkable case of path-integral control, we can recast the non-linear Bellman equation into a linear partial differential equation Kappen (2005b), Theodorou (2011) and Todorov (2006). This linear partial differential equation may then be estimated using a forward-time evolutionary process Doucet et al. (2001), Del Moral (2011) and Jefferies (2013). The evolutionary process targets the corresponding path integral in the Feynman-Kac lemma for the linear-transformed Bellman equation.

In this evolutionary process, the log-number of descendants in a particular state *g*, is then, in expectation, the optimal total reward (the value function) of the control problem. Since the value function is sufficient to reconstruct the optimal control, we can solve the problem entirely by taking expectations of this evolutionary process Theodorou et al. (2010). This biomimetic strategy for solving partial differential equations also appears in other fields e.g. particle filters for transfer-matrix methods in solid state physics Iba (2001).

### Entropy-regularized control on continuous time and discrete space

A control theorists therefore sees popgen as exactly this kind of stochastic solver. To be precise, popgen must be targeting the path integrals for a continuous time Markov jump process defined on genotypic space G with transitions governed by $\mu_{ij}$ i.e. a mutation process. We can infer that the controller in the target problem is able to exponentially-tilt the mutation rate into state $g^{\prime }$ by a factor $u_{g^{\prime }}(g)$, so that the controlled rate of transitioning from $g\to g^{\prime }$ is

( A2) $$\mu_{gg^{\prime }}^{\left(u\right)}=\mu_{gg^{\prime }}e^{u_{g^{\prime }}(g)}-\delta_{gg^{\prime }}\sum_{h}\mu_{gh}e^{u_{h}(g)}$$

The reward rate of the dual problem is equal to the fitness function of the genotypic state $f_{g}(t)$, minus a KL-Divergence rate which characterizes the cost of controlling the system

We derive the Bellman equation for the dual control problem. This extends the discrete time case from Todorov (2006) to continuous time Markov control.^1^. We apply the discrete space and time results of Todorov (2006) with transition function $p(g^{\prime }\;|\;g)=\delta_{gg^{\prime }}+\tau \mu_{gg^{\prime }}$ and take the formal limit τ→0.

#### Infinitessimal KL-divergence

Our continuous-time derivation of the appropriate cost deviates from Todorov (2006) in that instead of enforcing normalization with a lagrange multiplier, we start from a normalized description of continuous time KL-control, where the rows of the generator sum to zero, implying that the conditional probability generated sums to one. The diagonal terms $u_{gg}$ in the control cancel and are completely arbitrary:

( A3) $$\mu_{gg^{\prime }}^{\left(u\right)}=\mu_{gg^{\prime }}e^{u_{gg^{\prime }}}-\delta_{gg^{\prime }}\sum_{h}\mu_{gh}e^{u_{gh}}$$

The KL divergence rate can be calculated by the short-time Taylor expansion of the matrix exponential

( A4) $$\partial_{t}D_{KL}(p\|\pi )=\lim_{\tau \to 0}\partial_{\tau }\sum_{g^{\prime }}(\delta_{gg^{\prime }}+\tau \mu_{gg^{\prime }}^{\left(u\right)})\log\;\;\frac{\delta_{gg^{\prime }}+\tau \mu_{gg^{\prime }}^{\left(u\right)}}{\delta_{gg^{\prime }}+\tau \mu_{gg^{\prime }}}$$

( A5) $$=\sum_{g^{\prime }\neq g}\mu_{gg^{\prime }}^{\left(u\right)}\log\;\frac{\mu_{gg^{\prime }}^{\left(u\right)}}{\mu_{gg^{\prime }}}+\mu_{gg}^{\left(u\right)}-\mu_{gg}$$

since $\mu_{gg}=-\sum_{h\neq g}\mu_{gh}$

( A6) $$\partial_{t}D_{KL}(p\|\pi )=\sum_{g^{\prime }\neq g}\mu_{gg^{\prime }}^{\left(u\right)}\left(u_{gg^{\prime }}-1+e^{-u_{gg^{\prime }}}\right)$$

Thus the reward rate is

$$R_{g}(t\;|\;u)=f_{g}-\sum_{g^{\prime }\neq g}\mu_{gg^{\prime }}^{\left(u\right)}(e^{-u_{g^{\prime }}(g)}-(1-u_{g^{\prime }}(g)))$$

To summarize the derivation up until this point, *R* (and the terminal condition that control ends at t=T) is usually the “given,” the fundamental specification of the control problem. Instead, we have reconstructed this function by working backwards from the sampling algorithm (actually an evolutionary simulation).

With the reward function *R* in hand, its straightforward to optimize the control and construct the HJB equation. The change in the total optimal expected reward (backward in time)

( A7) $$\partial_{-t}\psi_{g}=\max_{u}R_{g}(t\;|\;u)$$

We determine the optimal control as a function of *ψ*

( A8) $$0=\underset{u=u^{*}}{|}\;\partial_{u_{gg^{\prime }}}\left(\;f_{g}+\sum_{g^{\prime }\neq g}\mu_{gg^{\prime }}^{\left(u\right)}\left(\psi_{g^{\prime }}-\psi_{g}+1-e^{-u_{gg^{\prime }}}-u_{gg^{\prime }}\right)\right)$$

this simplifies to

( A9) $$u_{gg^{\prime }}^{*}=\psi_{g^{\prime }}-\psi_{g}$$

with the HJB equation becoming

( A10) $$\partial_{-t}\psi_{g}=f_{g}+\sum_{g^{\prime }}\mu_{gg^{\prime }}\left(e^{\psi_{g^{\prime }}-\psi_{g}}-1\right)$$

The optimally controlled mutation operator is therefore ${mu}_{gg^{\prime }}^{\left(u\right)}=\mu_{gg^{\prime }}^{\psi }$, where,

( A11) $$\mu_{gg^{\prime }}^{\psi }\equiv \mu_{gg^{\prime }}e^{\psi_{g^{\prime }}-\psi_{g}}-\delta_{gg^{\prime }}\sum_{h}\mu_{gh}e^{\psi_{h}-\psi_{g}}$$

Comparing Equations (A11) and (12) jutifies the statement that ancestral lineages appear optimally controlled. This recapitualtes a remarkable result from (Kappen 2005b) that surviving paths generated by following the parenthood relationships and view them as paths forward in time appear to be sampled from the ensemble of optimally controlled trajectories.

#### Comparison with the pop-gen solution: missing discount term

When we compare Equations (A11) to (11), we see an important difference: the result above Equation (A11) does not include a discounting term. Inserting a discounting term $1/N_{e}\psi_{g}$ where $N_{e}=N/\lambda$ is a familiar modification of the HJB equation in control theory. A non-zero discount is essential for stability in reinforcement learning, with the discounting usually considered a necessary hyperparameter

However, a non-zero discount was *not* predicted by the path-integral control theory (PIC). Indeed, a discounting term breaks the assumptions upon which the path integral form depended, spoiling the linearity which allowed the use of the Feynman-Kac lemma. Our analysis from population genetics explain the emergence of a discount from population noise and can be seen as a finite-population modification of the Feynman-Kac lemma.

### HJB path integral and connection to forward evolutionary process

We can recast this equation as a linear equation via an exponential transformation.

( A12) $$\partial_{-t}e^{\psi_{g}}=f_{g}e^{\psi_{g}}+\sum_{g^{\prime }\neq g}\mu_{gg^{\prime }}\left(e^{\psi_{g^{\prime }}}-e^{\psi_{g}}\right)$$

( A13) $$\partial_{-t}e^{\psi_{g}}=\sum_{g^{\prime }}\left(\;f_{g}\delta_{gg^{\prime }}+\mu_{gg^{\prime }}\right)e^{\psi_{g^{\prime }}}$$

defining $e^{\psi_{g}}=z_{g}$ we recognize the linear quasispecies equation backward in time.

( A14) $$\partial_{-t}z_{g}=\sum_{g^{\prime }}\left(\;f_{g}\delta_{gg^{\prime }}+\mu_{gg^{\prime }}\right)z_{g^{\prime }}$$

This can be represented by the path integral

( A15) $$z_{g}(t)=\left\langle e^{\int_{t}^{T}f_{g(t^{\prime })}dt^{\prime }}|g(t)=g\right\rangle$$

with path density determined by the neutral mutation generator *μ* and starting from *g*. By construction, *z* can be estimated up to a multiplicative constant via an evolutionary algorithm, where in the infinite population size limit, the average number of descendants of individuals in a genotypic class *g*, alive at time *T* is proportional to $z_{g}(t)$.

#### The path integral

Inserting a discrete time mutational operator at ever-finer intervals, we see that the number of descendants can be expressed as a path integral generated by the continuous mutational process,

( A16) $$w_{g}(t,T)=\lim_{\epsilon \to 0}\sum_{g(t)\ldots g(T)}[e^{\epsilon \;\mu }{]}_{g(t)}^{g(t+\epsilon )}e^{\epsilon \left(\beta_{g(t+\epsilon )}-\delta_{g(t+\epsilon )}\right)}$$

( A17) $$\begin{array}{rl} & \;\times [e^{\epsilon \;\mu }{]}_{g(t+\epsilon )}^{g(t+2\epsilon )}e^{\epsilon (\beta_{g(t+2\epsilon )}-\delta_{g(t+2\epsilon )})}\\ & \;\cdots \times [e^{\epsilon \;\mu }{]}_{g(T-\epsilon )}^{g(T)}e^{\epsilon (\beta_{g(T)}-\delta_{g(T)})} \end{array}$$

Where $[e^{\epsilon \;\mu }{]}_{g(T-\epsilon )}^{g(T)}$ is the matrix exponential, (the short time propagator for the neutral mutation process).

#### Effect of discounting on path-integral control

We find that we can account for genetic drift of finite population sizes by adding a discount rate *γ* to the control problem. In Todorov (2009) discounting is treated as a modification of the (in that reference, discrete-time) equation for *z*.

$$z_{g}(t)=\sum_{g^{\prime }}\left(\delta_{gg^{\prime }}+\tau \left(\;f_{g}\delta_{gg^{\prime }}+\mu_{gg^{\prime }}\right)\right)z_{g}(t+\tau {)}^{1-\gamma \tau }$$

In the continuous limit this yields the modification,

( A18) $$\partial_{-t}\psi_{g}=f_{g}+\sum_{g^{\prime }}\mu_{gg^{\prime }}e^{\psi_{g^{\prime }}-\psi_{g}}-\gamma \psi_{g}$$

where we have determined that the discounting time scale and the coalescent time scale should match $\gamma =1/N_{e}$. Thus the optimal control remains the same, only the Bellman equation becomes modified by a decay factor.

## Appendix B: Coordinate transform of the Kimura operator

The mutation operator $L_{x}^{\mathrm{mut}}=\sum_{hh^{\prime }}(x_{h^{\prime }}\mu_{h^{\prime }h}-x_{h}\mu_{hh^{\prime }})\partial_{h}$

( B1) $$\frac{e^{-\Psi_{g}(x)}}{x_{g}}L_{x}^{\mathrm{mut}}x_{g}e^{\Psi_{g}(x)}=\sum_{g^{\prime }}\left(\frac{x_{g^{\prime }}}{x_{g}}\mu_{g^{\prime }g}-\mu_{gg^{\prime }}\right)+L_{x}^{\mathrm{mut}}\Psi_{g}(x)$$

and $L_{x}^{\mathrm{sel}}=(f_{h}-\sum_{h^{\prime }}x_{h}^{\prime }f_{h}^{\prime })\partial_{h}$

( B2) $$\frac{e^{-\Psi_{g}(x)}}{x_{g}}L_{x}^{\mathrm{sel}}x_{g}e^{\Psi_{g}(x)}=f_{g}-\sum_{g^{\prime }}x_{g^{\prime }}f_{g^{\prime }}+L_{x}^{\mathrm{mut}}\Psi_{g}(x)$$

and finally $L_{x}^{\mathrm{drift}}=\frac{1}{2N_{e}}\sum_{hh^{\prime }}(x_{h}(\delta_{hh^{\prime }}-x_{h^{\prime }}))\partial_{h^{\prime }}\partial_{h}$

( B3) $$\frac{e^{-\Psi_{g}(x)}}{x_{g}}L_{x}^{\mathrm{drift}}x_{g}e^{\Psi_{g}(x)}=\frac{1}{N_{e}}\left(\frac{\partial \Psi_{g}}{\partial x_{g}}-\sum_{h^{\prime }}x_{h^{\prime }}\frac{\partial \Psi_{g}}{\partial x_{h^{\prime }}}\right)+L_{x}^{\mathrm{drift}}(\Psi_{g})$$

( B4) $$\;+\;\frac{1}{2N_{e}}\left[\sum_{h}x_{h}{\left(\frac{\partial \Psi_{g}}{\partial x_{h}}\right)}^{2}-{\left(\sum_{h}x_{h}\frac{\partial \Psi_{g}}{\partial x_{h}}\right)}^{2}\right]$$

The end result of the coordinate transformation is

( B5) $$-\partial_{t}\Psi_{g}(x)=\sum_{g^{\prime }}\mu_{gg^{\prime }}\left(e^{\Psi_{g^{\prime }}(x)-\Psi_{g}(x)}-1\right)+f_{g}-\sum_{g^{\prime }}x_{g^{\prime }}f_{g^{\prime }}+L_{x}\Psi_{g}(x)$$

( B6) $$\;+\frac{1}{N_{e}}\left(\frac{\partial \Psi_{g}}{\partial x_{g}}-\sum_{h^{\prime }}x_{h^{\prime }}\frac{\partial \Psi_{g}}{\partial x_{h^{\prime }}}\right)$$

( B7) $$\;+\frac{1}{2N_{e}}\left[\sum_{h}x_{h}{\left(\frac{\partial \Psi_{g}}{\partial x_{h}}\right)}^{2}-{\left(\sum_{h}x_{h}\frac{\partial \Psi_{g}}{\partial x_{h}}\right)}^{2}\right]$$

## Appendix C: Gauge freedom and frequency-decoupling

Substituting the decomposition $\Psi_{g}(x)=\psi_{g}-\phi (x)$ into the exact fitness potential equation yields the full population-coupled dynamics:

( C1) $$\begin{array}{rl} -\partial_{t}\psi_{g}-\partial_{t}\phi (x) & =\sum_{g^{\prime }}\mu_{gg^{\prime }}\left(e^{\psi_{g^{\prime }}-\psi_{g}}-1\right)+f_{g}-\sum_{g^{\prime }}x_{g^{\prime }}f_{g^{\prime }}\\ & \;+L_{x}\phi (x)+\frac{1}{N_{e}}\left(\frac{\partial \phi (x)}{\partial x_{g}}-\sum_{h}x_{h}\frac{\partial \phi (x)}{\partial x_{h}}\right)\\ & \;+\frac{1}{2N_{e}}\left[\sum_{h}x_{h}{\left(\frac{\partial \phi (x)}{\partial x_{h}}\right)}^{2}-{\left(\sum_{h}x_{h}\frac{\partial \phi (x)}{\partial x_{h}}\right)}^{2}\right] \end{array}$$

To extract the evolution of ψ, we isolate the *g*-dependent terms on each side of the equation

( C2) $$-\partial_{t}\psi_{g}(t)=\sum_{g^{\prime }}\mu_{gg^{\prime }}\left(e^{\psi_{g^{\prime }}-\psi_{g}}-1\right)+f_{g}-\frac{1}{N_{e}}\frac{\partial \phi (x)}{\partial x_{g}}.$$

Exact separation is obstructed by the frequency-dependent Ito-drift term, $\frac{1}{N_{e}}\frac{\partial \phi (x)}{\partial x_{g}}=\frac{1}{N_{e}}e^{\psi_{g}-\phi (x)}$.

The full potential $\Psi_{g}(x)$ and the observable lineage dynamics are invariant under an allele-independent scalar shift applied to $\psi_{g}$, yielding $\psi_{g}\to \psi_{g}+\xi (x)$. Because the log-normalization term ϕ(x) is determined by the probability conservation constraint, this shift algebraically factors out of the logarithm:

( C3) $$\phi (x)\to \log\;\left(\sum_{g}x_{g}e^{\psi_{g}+\xi (x)}\right)=\log\;\left(e^{\xi (x)}\sum_{g}x_{g}e^{\psi_{g}}\right)=\xi (x)+\phi (x)$$

Consequently, the arbitrary shift ξ(x) cancels exactly in the difference $\Psi_{g}(x)=\psi_{g}-\phi (x)$. This exact gauge invariance is the time-integrated equivalent of the standard replicator invariance under shifts in absolute fitness, $f_{g}(x)\to f_{g}(x)+C(x)$. It represents the reproductive-value equivalent of the familiar principle that absolute fitness is unobservable; only relative fitness differences govern observable frequency dynamics.

We use this gauge freedom to minimize the frequency-dependent coupling of $\psi_{g}$. Taylor expanding the Ito-drift term gives:

( C4) $$\frac{1}{N_{e}}e^{\psi_{g}-\phi (x)}=\frac{1}{N_{e}}\left[1+\psi_{g}-\phi (x)+O\left((\psi_{g}-\phi (x){)}^{2}\right)\right]$$

Selecting the specific gauge shift $\xi (x)=\frac{1}{N_{e}}(1-\phi (x))$ absorbs the shared frequency-dependence at linear order. This reduces the *g*-dependent Ito drift to $\frac{1}{N_{e}}\psi_{g}+O(\frac{1}{N_{e}}(\psi_{g}-\phi (x){)}^{2})$, yielding the first-order decoupled equation:

( C5) $$-\partial_{t}\psi_{g}(t)=\sum_{g^{\prime }}\mu_{gg^{\prime }}\left(e^{\psi_{g^{\prime }}-\psi_{g}}-1\right)+f_{g}-\frac{1}{N_{e}}\psi_{g}$$

This isolates the lineage-specific dynamics to first order, establishing the decoupled system we use for inference.

### Convexity and inference bias under strong selection

If the population has a fixed genotype $g_{0}$ then $\phi (x)=\psi_{g_{0}}$. ϕ(x) can therefore be interpreseted as representing the background fitness potential of the population. Deviations from this background define the selective regime: $\psi_{g}>\phi (x)$ indicates positive selection, while $\psi_{g}<\phi (x)$ indicates negative selection. By linearizing the frequency-dependent Ito-drift term $\frac{1}{N_{e}}e^{\psi_{g}-\phi (x)}$ to achieve the decoupled ODE Equation (C5), we systematically introduce an inference bias for regimes where the weak-selection assumption breaks down.

This bias is arises from the convexity of the exponential function. Expanding the drift term to second order yields:

( C6) $$\frac{1}{N_{e}}e^{\psi_{g}-\phi (x)}\approx \frac{1}{N_{e}}\left(1+(\psi_{g}-\phi (x))+\frac{1}{2}(\psi_{g}-\phi (x){)}^{2}\right)$$

The weak-selection approximation truncates this expansion at linear order. The exponential function is strictly convex and the omitted quadratic term $\frac{1}{2N_{e}}(\psi_{g}-\phi (x){)}^{2}$ is always non-negative.

Consequently, the linear approximation systematically underestimates the drift term. The true dynamics therefore contain an additional non-linear penalty, or “drag,” on the fitness potential that is absent in the decoupled linear model. This omission drives opposite biasing behaviors during parameter inference depending on the sign of selection:

- Attenuation of positive selection: For a positively selected allele ($\psi_{g}>\phi (x)$), the true evolutionary process exerts a stronger decay on the fitness potential than the linear model predicts. Because the linear model lacks this quadratic drag, it will overestimate the fitness potential generated by a given fitness parameter $f_{g}$. To match the observed phylogenetic data, the inference framework must therefore systematically underestimate $f_{g}$. Thus, the inferred selection coefficient acts as a conservative lower bound for positive selection.
- Enhancement of negative selection: For a negatively selected allele ($\psi_{g}<\phi (x)$), the linear approximation $1+(\psi_{g}-\phi (x))$ decreases linearly, whereas the true exponential term remains bounded above zero. The linear model predicts an artificially rapid decay of the fitness potential backward in time. To match the less severe decay actually observed in the survival of deleterious lineages, the inference framework will compensate by overestimating the magnitude of the negative selection coefficient $f_{g}$.

## Appendix D: Inference-methods and tree likelihood

### Interactions between fitness potential of contemporaneous lineages

In the weak-selection limit, we can leverage the frequency independence to dramatically reduce the effort required to evaluate the likelihood of genotypic data. Instead of using Monte Carlo sampling to estimate the integral over *X*, we can analytically marginalize out these hidden paths.

We begin by separating the probability of the tree topology and the probability of the data, given the tree.

( D1) P(G,T|Θ)=P(T|Θ)×P(G|T,Θ)

Our parameters are those neccesary for specifying the Kimura operator the (potentially time-dependent) drift, mutation, fitness functions $\Theta =N_{e},\mu ,f$.

To apply our weak-selection results for this problem, we must determine how coalescence affects the fitness potential *ψ*. For two lineages *i* and *j* to coalesce in a short time *τ*, one of the $Nx_{g}\beta_{g}\tau$ coalescent event must occur on exactly this pair from the (Nxg2) possible *g*-lineages in the population.

( D2) $$\left[ij\text{-coalescent hazard for}\;w_{g}^{i}\right]=w_{g}^{i}w_{g}^{\;j}\frac{2\beta_{g}}{Nx_{g}}$$

since we are concerned with the weak selection limit anyway, we can replace $2\beta_{g}/N=1/N_{e}$, ignoring terms of size $\frac{\;f_{g}-\sum_{h}x_{h}f_{h}}{\lambda }$. Using the same weak selection and gauge choice as in the single lineage case

( D3) $$\left[\text{coalescent decay of}\;\psi_{g}^{i}\right]=\sum_{\;j\neq i}\frac{1}{N_{e}}e^{\psi_{g}^{j}-\phi^{j}(x)}-(1-\phi^{j}(x))$$

( D4) $$\approx \sum_{\;j\neq i}\frac{1}{N_{e}}\psi_{g}^{j}$$

Including this interaction term into Equation (11) results in an equation for the evolution of $\psi^{i}$ along each of the branches. These coalescent interactions now enforce an enhanced decay along each of the branches *i* that coexist with other branches *k* at time *t*.

( D5) $$-\partial_{t}\psi_{g}^{i}=\sum_{g^{\prime }}\mu_{gg^{\prime }}\left(e^{\psi_{g^{\prime }}^{i}-\psi_{g}^{i}}-1\right)+f_{g}-\frac{1}{N_{e}}\sum_{k}\psi_{g}^{k}$$

At the moment of coalescence C(i)→i, we have the boundary condition at $T_{i}$

( D6) $$\psi_{g}^{i}=\left\{ \begin{array}{cc} \sum_{\;j\in C(i)}\psi_{g}^{\;j}\; & \text{\textbackslash{},for edge}\;i\;\text{with children}\;C(i)\\ 0 & \text{\textbackslash{},for edge}\;i\;\text{terminating in an observation} \end{array}\right.$$

This decay term can be understood as the observed lack of coalescence drives decoherence in the fitness potentials of contemporaneous lineages.

#### Tree likelihood is neutral at same order

The tree likelihood is determined by the coalescent hazard $\Lambda_{ij}(t)$ for contemporaneous lineages *i j*, exactly as a Poisson process with time varying rates, with rate

( D7) $$\log\;P(T\;|\;N_{e},\mu ,f)=\sum_{ij}\int_{t}\Lambda_{ij}(t)+\sum_{c}\log\;\Lambda_{c_{1}c_{2}}(t_{c})$$

where the second sum is over the observed coalescent events *c* between lineages $c_{1}$ and $c_{2}$ occuring at time $t_{c}$. To account for the fact that lineages must share a type to coalesce, making the hazard

( D8) $$\Lambda_{ij}=\sum_{g}\frac{w_{g}^{i}w_{g}^{\;j}}{N_{e}x_{g}}$$

( D9) $$=\sum_{g}\frac{x_{g}e^{\Psi_{g}^{i}(x)+\Psi_{g}^{\;j}(x)}}{N_{e}}$$

assuming the self-consistent decomposition as before $\Psi_{g}^{i}(x)=\psi_{g}-\phi (x)$, we have, to second order

( D10) $$\begin{array}{rl} & \approx \frac{1+2{\mathrm{cov}}_{x}(\psi^{i},\psi^{\;j})}{N_{e}}=\frac{1}{N_{e}}\\ & \;+O(\frac{(\psi_{g}-\phi (x){)}^{2}}{N_{e}}) \end{array}$$

In the weak selection limit, the frequency dependence of the tree likelihood vanishes, and luckily the coalescent hazard appears neutral at the same approximation order as the weak selection result, $O({N_{e}}^{-1}(\psi_{g}-\phi (x){)}^{2})$. In this case the tree likelihood is only a function of the effective population size $N_{e}$. For coalescent times $t_{c}$,

( D11) $$\log\;P(T\;|\;N_{e},\mu ,f)=\log\;P(T\;|\;N_{e})$$

( D12) $$=\sum_{ij}\int_{t}\frac{\left[i\;\text{and}\;j\;\text{alive at}\;t\right]}{N_{e}(t)}-\sum_{c}\log\;N_{e}(t_{c})$$

with [X] the Iverson bracket, 1, if X is true and 0 if X false. The cancelation in the denominator $x_{g}$, reflects the fact that, under weak selection, tree structure is unaffected by allelic diversity and mutations can be trivially painted on a neutrally coalescent tree.

### Observation likelihood from biased-mutational operator

Given functions $f_{g}$ and $\mu_{gg^{\prime }}$ and $N_{e}$, these define via Equation (D6) a time dependent fitness potential over the genotypic state space at each point along along each edge *i* of this tree Equation (D6) $\psi_{g}^{i}(t)$, and a biased mutational operator $\mu^{\psi }(t)$ for times between the lineage coalescence $t_{i}$ and its termination in an observation or birth event at $T_{i}$. The biased mutational operator $\mu^{\psi }$ then determines a conditional probability density for an initial genotype *g* to evolve into a new genotype $g^{\prime }$ by the end of the lineage exactly analogous to Felsenstein’s pruning algorithm Felsenstein (1981) and Bouckaert et al. (2014),

( D13) $$p_{gg^{\prime }}^{i}={\left[\exp\;\int_{t_{i}}^{T_{i}}\mu^{\psi }(t^{\prime })dt^{\prime }\right]}_{gg^{\prime }}.$$

where exp∫⋅dt is the time-ordered matrix exponential. The likelihood of observing a tree rooted on lineage *i* starting in state *g* is defined recursively

( D14) $$L_{g}^{i}=\left\{ \begin{array}{cc} \sum_{g^{\prime }}p_{gg^{\prime }}^{i}\prod_{\;j\in C(i)}L_{g^{\prime }}^{j}\; & \text{\textbackslash{},for edge}\;i\;\text{with children}\;C(i)\\ p_{gG_{i}}^{i} & \text{\textbackslash{},for edge}\;i\;\text{terminating in}\;G_{i} \end{array}\right.$$

#### Common ancestor prior

We must choose an averaging scheme over the final common ancestor state. We propose the stationary distribution of the ancestral process at the root time as a natural prior $\pi_{g}^{\psi }$ of $\mu^{\psi }$ which is easy to compute.

( D15) $$\pi_{g}^{\psi }=\frac{\pi_{g}e^{\psi_{g}(t_{0})}}{\sum_{h}\pi_{h}e^{\psi_{h}(t_{0})}}$$

Then the total probability of the observations given the tree is

( D16) $$P(G\;|\;T,N_{e},\mu ,f)=\sum_{g}\pi_{g}^{\psi }L_{g}^{\mathrm{root}}$$

Other choices, such as a uniform prior, are also possible.

### Exact inference using the lineage frequencies and marginalization over paths

For contrast, we briefly describe the procedure to construct the exact likelihood given a particular frequency history *X*, a path through frequency space governed by the Fisher-Kimura stochastic generator $L^{†}$.

( D17) $$P(G,T\;|\;\Theta )=\underset{X\;|\;L^{†}(\Theta )}{E}\;P(G\;|\;X,\Theta )\times P(T\;|\;G,X,\Theta )$$

Here, $P(G\;|\;X,\Theta )=\prod_{i}x_{G_{i}}(T_{i})$ represents multinomial sampling at each time point.

With each proposal path we can evaluate the tree likelihood P(T|G,X,Θ) using the *forward* (but still time reversed, still from tip to root!) Kolmogorov equation with $\mu^{\mathrm{rev}}(x)$ for each lineage w with coalescnce hazard described above

( D18) $$\left[ij\text{-coalescent hazard for}\;w_{g}^{i}\right]=w_{g}^{i}w_{g}^{\;j}\frac{2\beta_{g}}{Nx_{g}}$$

With sophisticated importance samplers for *X*, this exact likelihood might become feasible for small systems and few time points.

## Appendix E: The birth-death noise structure

Recent state-dependent birth-death models Lewinsohn et al. (2023) explicitly modify forward transition rates by the ratio of sampling probabilities ($\frac{P_{j}}{P_{i}}$), an operation mathematically equivalent to the optimal control policy derived in our framework ($\mu e^{\Delta \Psi }$). A tree likelihood could theoretically be computed using the raw backward Kolmogorov equation, but these methods take an extra step of conditioning the forward generator on eventual sampling, finding that this conditioned process yields a more stable likelihood for inference. Our work provides the theoretical justification for this practice of using the forward equation: the raw likelihood is inappropriate for inference because it comes from the wrong (unconstrained, unconditioned) model. But the forward propagator is equivalent to a “birth-death potential” that steers lineages away from extinction, and is quite similar to our fitness potential.

However, this birth-death potential has distinct dynamics. We consider a standard multi-type birth-death process conditioned on a single sampling event. Let $E_{g}(t)$ be the probability that a single lineage currently in state *g* at time *t* will not be sampled (i.e. goes extinct or remains unsampled) before the present time *T*.

In standard birth-death-sampling models, this probability evolves according to the backward Kolomolgorov birth-death equation:

( E1) $$-\frac{dE_{g}}{dt}=\beta_{g}(E_{g}^{2}-E_{g})+\delta_{g}(1-E_{g})+\sum_{g^{\prime }}\mu_{gg^{\prime }}E_{g^{\prime }}$$

where $\beta_{g}$, $\delta_{g}$, and $\mu_{gg^{\prime }}$ are the birth, death, and mutation generator respectively. We define the survival probability, $P_{g}(t)=1-E_{g}(t)$ that the lineage is sampled. This closure is only possible in the case of a single sample event, otherwise there are two separate equations. Substituting $E_{g}=1-P_{g}$ into the Riccati equation and simplifying using the relations for net fitness $f_{g}-\bar{f}(x)=\beta_{g}-\delta_{g}$,

( E2) $$-\frac{dP_{g}}{dt}=(f_{g}-\bar{f}(x))P_{g}-\beta_{g}P_{g}^{2}+O(\mu )$$

We apply the logarithmic transformation to map this probability to a fitness potential $\Psi_{g}^{BD}=\log\;NP_{g}$. The boundary condition at sampling time *T* for a single identified lineage is $P_{g}(T)=1/N$, implying that $\Psi_{g}^{BD}(T)=0$ matching the fitness potential boundary condition.

We can therefore rewrite the non-linear decay term

( E3) $$-\frac{d\Psi_{g}^{BD}}{dt}\;=\;\frac{1}{P_{g}}\left(-\frac{dP_{g}}{dt}\right)=f_{g}-\frac{\beta_{g}}{N}e^{\Psi_{g}^{BD}}$$

The effective population size in terms of the noise intensity is $N_{e}=N/\lambda$. Assuming weak selection (f≪λ), the birth rate is dominated by turnover, $\beta_{g}\approx \lambda /2$. We can therefore rewrite the non-linear decay term:

( E4) $$\frac{\beta_{g}}{N}(e^{\Psi_{g}^{BD}}-1)\approx \frac{\lambda }{2}\cdot \frac{1}{N}\Psi_{g}^{BD}=\frac{1}{2N_{e}}\Psi_{g}^{BD}$$

Substituting this back into the evolution equation:

( E5) $$-\frac{d\Psi_{g}^{BD}}{dt}\approx f_{g}-\bar{f}-\frac{1}{2N_{e}}\Psi_{g}^{BD}$$

Comparing this result to the fitness potential derived from the Wright-Fisher process Equation (11), which contains a decay term of $1/N_{e}$, we see a discrepancy of a factor of 1/2 in the decay term despite them targeting the same continuum process. This birth-death potential decay term is in contradiction with numerical experiments and the analytic results for the rare-mutation steady-state limit of a biallelic system (see Ancestral stationary distribution of a biallelic system).

## Appendix F: Additional models

### Triallelic system

For completeness we consider more complicated systems beyond the biallelic case. Since mutations are relatively rare, the fitness potential exponentially decays backward in time. We sample the population at time at $2N_{e}$ and then imagine we can reconstruct the population tree with perfect fidelity. We trace a lineage backward in time and record the genotypic changes. We compare these observations to the predictions from the optimal control result on the dual control problem as shown in Fig. A1.

In this simulation we consider three genotypes of differing fitnesses. The fitness potential is the time integral of these fitness differences backward in time.

( F1) $$f_{g}=a_{g}\;\text{with}\;\left\{ \begin{array}{c} a_{1}=2\times N_{e}^{-1}\\ a_{2}=0\times N_{e}^{-1}\\ a_{3}=-1\times N_{e}^{-1} \end{array}\right.$$

### Breakdown of theory with strong positive selection and low mutation rates

At the mutational level there is a breakdown in the symmetry of the mutation rate bias around the neutral value. When plotted on a log scale we can see that even for the case where the breakdown is worst, there is a significant amount of bias in the amount of positive selection.

Suppose that a mutation is positively selected to the extent that every time there is a mutation in the population, it reaches fixation. The rate of such events occurring is only μN, clearly the maximum control on a positively selected allele is $\delta \psi_{\max}=\log\;N$. On the other hand, for negative selection, there is no limit to how often we can reject a given mutation. This kind of breakdown can therefore be seen as a result of the discrete nature of the population relative to the continuum limit of the Kimura equation itself.

## Appendix G: Simulation details

Simulations were performed in Julia Bezanson et al. (2017) using a balanced Moran process with units N=1000, and λ=1 giving $N_{e}=1000$ in some arbitrary units of time (in the seasonal model this is in days). The inference problem was simulated N=2000 in the hope that this would lead to less biased results than N=1000 (it didn’t). Unless otherwise specified the mutations are set so that the unitless parameter, θ=0.1. Lower *θ* leads to larger deviations from the constant $N_{e}$ theory due strong correlation in the fates of individual mutations and the average fitness of the population. The fitness potential differential equations were solved using the DifferentialEquations.jl package Rackauckas and Nie (2017), MLE optimization was performed with the BOBYQA algorithm, Powell (2009) using the package PRIMA.jl, Zhang (2023), plots made with Makie.jl Danisch and Krumbiegel (2021).
