# Supplementary material for: Optimal control and the dynamics of ancestral lineages
Source: Genetics. 2026 May 18;233(4):iyag122. doi: 10.1093/genetics/iyag122 (PMC13594677; doi:10.1093/genetics/iyag122)
Supplement: Supplementary file 1 [file iyag122_Peer_Review_History.pdf]

GENETICS

Optimal Control and the Dynamics of Ancestral Lineages

Colin LaMont

NOTE: The reviews and decision letters are unedited and appear as submitted by the reviewers.
In extremely rare instances and as determined by a Senior Editor or the EIC, portions of a review may be redacted. If a review is signed, the reviewer has agreed to no longer remain anonymous.
The review history appears in chronological order.

Review Timeline:

Submission Date:	2025-12-03
Editorial Decision:	2026-01-31
Resubmission Received:	2026-03-28
Accepted:	2026-04-29

January 31, 2026

GENETICS-2025-308846
Optimal Control and the Dynamics of Ancestral Lineages

Dear Dr. LaMont:

An expert in the field has reviewed your manuscript, and I have read it as well.

The reviewer enjoyed this MS and has made several suggestions to strengthen it which include a more detailed explanation of technical terms and main equations. You can read their review at the end of this email.

I also found this MS interesting as it brings new ideas to popgen theory. But if I understand correctly, these ideas are most helpful in the weak selection regime (as eqn 2 simplifies to give eqn 12) but otherwise the problem can be tackled only numerically (as discussed briefly on p.9). On p. 17 in `Weak selection' section, this point is discussed but only briefly and I found this discussion to be somewhat vague - specifically, what is meant by "suitable" averaging? "often" decomposes? "arbitrarily" strong selection is immediately followed by "how strong is too strong". I think a much clearer and more precise discussion would help the reader who wishes to skip the technical sections. But perhaps you can refer to equation numbers for the mathematically-inclined in the discussion section.

Regarding references: On first 2 lines of Conclusion section, it is stated that "a link between control theory and evolution has been previously proposed". I checked Kappen's review [ref 26] but did not find any such discussion - please elaborate this statement and/or correct the reference. In Sec. 5.1.3, related work of David Saakian and colleagues [see, for e.g., Journal of Statistical Physics, Vol. 128, No. 3, August 2007] who applied the Hamilton-Jacobi formalism to understand stationary state properties in quasispecies models should also be mentioned.

While your manuscript is not currently acceptable for publication in GENETICS, we would welcome a substantially revised manuscript.

We look forward to receiving your revised manuscript. Please let the editorial office know approximately how long you expect to need for revisions.

Upon resubmission, please include:
1. A clean version of your manuscript;
2. A marked version of your manuscript in which you highlight significant revisions carried out in response to the major points raised by the editor/reviewers (track changes is acceptable if preferred);
3. A detailed response to the editor's/reviewers' feedback and to the concerns listed above. Please reference line numbers in this response to aid the editor and reviewers.

Your paper will likely be sent back out for review.

Additionally, please ensure that your resubmission is formatted for GENETICS
https://academic.oup.com/genetics/pages/general-instructions

Follow this link to submit the revised manuscript: Link Not Available

Sincerely,

Kavita Jain
Associate Editor
GENETICS

Approved by:
Nick Barton
Senior Editor
GENETICS

Reviewer #1 :

I very much enjoyed reading this paper from LaMont, which draws a link between quantitative methods from optimal control, statistical physics, and population genetics. Beyond a proposed mathematical relationship, the author also uses these ideas from optimal control to develop an approach for inferring selection from phylogenetic trees. To my knowledge, this particular perspective is novel, and I believe that this work will be of special interest to theorists.

There are some places where the manuscript could be clarified and improved. Given the inspiration drawn from different quantitative traditions (which is a good thing), some parts of the paper might be more difficult to follow even for a technical audience.

Main comments

1. One part of the argument that I don't completely follow is the passage from eq. 10 to eq. 11, which relies in part on a choice of gauge to simplify the equations. The choice of gauge doesn't seem to be described in the SI, and, at least to this reader, it is not entirely obvious. This calculation could be described in more detail in the SI. The meaning seems fairly transparent, but it could be helpful to be even a bit more explicit here with definitions, since the term "gauge freedom" might not be broadly understood.

2. At the top of p. 6, there's an assumption that $\Psi_g(x) = \psi_g - \phi(x)$, i.e., that the frequency dependence of Ψ can be contained in a separate term ϕ. This assumption is important for the remaining calculations, but it isn't closely examined in the text. What does this mean physically or biologically?

3. One of the interesting aspects of this work is the connection between optimal control and evolutionary theory. There are a number of places where the author describes a control theory interpretation of a selection event. As a metaphor, this is interesting, but I wonder whether this mathematical relationship has deeper consequences. Could the author comment, even hypothetically, on further insights that could be enabled by the parallel between control and population genetics?

4. In the Discussion, the author makes a nice comparison between the approach proposed in the current paper and existing tree- or trajectory-based inference approaches. There, the comment that trajectory-based approaches rely only on marginal allele frequencies does not appear to be entirely correct. At least in some approaches, including some cited here, the full population path integral (mentioned at the end of section 5.1.3) is employed. There are of course drawbacks to these approaches - they require frequent samples in time, and they also employ typically approximations like weak selection - but by considering the evolution of the full genotype distribution across time, they can address this specific and important concern. However, the present paper also offers a complementary approach to this challenge.

Associate Editor Comments:

Response to the Associate Editor

Editor Comment: But if I understand correctly, these ideas are most helpful in the weak selection regime (as eqn 2 simplifies to give eqn 12) but otherwise the problem can be tackled only numerically (as discussed briefly on p.9). On p. 17 in 'Weak selection' section, this point is discussed but only briefly and I found this discussion to be somewhat vague - specifically, what is meant by "suitable" averaging? "often" decomposes? "arbitrarily" strong selection is immediately followed by "how strong is too strong". I think a much clearer and more precise discussion would help the reader who wishes to skip the technical sections. But perhaps you can refer to equation numbers for the mathematically-inclined in the discussion section.

Response: We thank the Editor for pointing out the vague phrasing in this important section. We have rewritten the section "The limits of the weak-selection approximation" to provide a precise explanation of how the method behaves when the weak-selection assumption breaks down.

We explicitly outline how the convexity of the exponential drift term leads to systematic, directional biases, providing a conservative lower bound for positive selection, and and also a lower bound on negative selection (that is negative selection is inferred to be even more negative).

The full fitness potential $\Psi(\mathbf{x}, t, T)$ determines ancestral lineage dynamics via Equation 8. Ψ depends on $\mathbf{x}$ and general phylogenetic likelihoods require integration over the unobserved population history $\mathbf{x}(t)$. Under the separable decomposition $\Psi_g \approx \psi_g - \phi(\mathbf{x})$ the $\mathbf{x}$-dependence drops out of the dynamical equations. Because this decomposition is derived in the weak-selection limit, establishing its domain of validity is necessary for applications where selection is strong ($\sigma \gg 1$).

Exactness at the null hypothesis ($\sigma = 0$) ensures that the framework is a valid tool for selection detection, even as quantification of σ is approximate. The site-specific likelihood (Equation 28) from linked neutral components naturally provides a reference distribution for a phylogeny-aware likelihood-ratio test.

For selection quantification, the weak-selection approximation introduces a bias in the inferred selection coefficients $\sigma = 2N_e\Delta f$ when selection is strong (see Appendix: Convexity and inference bias under strong selection) The approximation has discarded the non-linear component of a drift-induced drag on leaving descendants. For positively selected alleles, the result is that we underestimate the fitness advantage required to drive observed dynamics, and the inferred σ is a conservative lower bound for positive selection. Conversely, the approximation is expected to overestimate the magnitude of strong negative selection.

The structural reliability of this approximation is supported by its exactness at temporal extremes. Over short timescales, the variation in the fitness potential remains small, rendering the decomposition locally exact (see the discussion after Equation 11). On long timescales, the weak limit recovers the exact ancestral stationary distribution for mutation-selection balance in the rare-mutation limit (provided the mutational operator obeys detailed balance; see Ancestral stationary distribution of a biallelic system).

Although the approximation outputs an interpretable lower bounds on σ in the presence of strong allele-dependent selection, even weak frequency-dependent fitness (e.g. [23?]) breaks the separability assumption $\Psi \approx \psi_g - \phi(\mathbf{x})$ at leading order (Equation 10). Intuitively, $\mathbf{x}$-conditioning cannot be avoided if we wish to model $\mathbf{x}$-dependent changes in relative fitness. To mathematically describe this bias in detail, we have also added a new subsection to the Appendix, Convexity and inference bias under strong selection (p. 16 lines 32-72):

Editor Comment: Regarding references: On first 2 lines of Conclusion section, it is stated that 'a link between control theory and evolution has been previously proposed'. I checked Kappen's review [ref 26] but did not find any such discussion - please elaborate this statement and/or correct the reference.

> Response: Yes, this citation was incorrect. Eq. 12 from [11] only establishes a rudimentary version of a particle filter (no resampling), and the connection to evolution is not explicit. Kappen, et al., fully develops the connection to more sophisticated particle filters in a later paper [17]. The link between particle filters and biologcal evolution traces to [10] and the link to lineage sampling in particular can be found in Ch. 11 of [4].
>
> We have filled in these gaps and are explicit that Kappen only establishes the link to the particle filter. A link between control theory and evolution has previously emerged. Particle filters, algorithms that mimic Darwinian evolution [10, 4], can be used to solve an optimal control problem [12, 17].

Editor Comment: In Sec. 5.1.3, related work of David Saakian and colleagues [see, for e.g., Journal of Statistical Physics, Vol. 128, No. 3, August 2007] who applied the Hamilton-Jacobi formalism to understand stationary state properties in quasispecies models should also be mentioned.

> Response: We thank the Editor for highlighting this important literature. We have integrated Saakian's work into our discussion on quasispecies theory and the Hamilton-Jacobi formalism. We specifically note how logarithmic transformations allow for exact equilibrium distributions and perturbation theories. This addition reads (p.9 lines 102-112):
>
> In the infinite population limit ($N_e \to \infty$), the exponentiated value function $z_g = e^{\psi_g}$ follows a linear operator equivalent to the quasispecies equation, $\partial_t \mathbf{z} + (\mu + \mathbf{f})\mathbf{z} = 0$. This connection is a consequence of the Feynman-Kac lemma, which relates the stochastic Bellman equation to a linear path integral. Therefore, a logarithmic transformation of the quasispeces density yields a forward-time Hamilton-Jacobi equation mathematically close to Equation 11 [1, 6, 2, 16]. Through this logarithmic transformation, it is possible to derive equilibrium results and perturbation theories for complex fitness landscapes [5, 18, 20, 19]

Reviewer Comment: One part of the argument that I don't completely follow is the passage from eq. 10 to eq. 11, which relies in part on a choice of gauge to simplify the equations. The choice of gauge doesn't seem to be described in the SI, and, at least to this reader, it is not entirely obvious. This calculation could be described in more detail in the SI. The meaning seems fairly transparent, but it could be helpful to be even a bit more explicit here with definitions, since the term 'gauge freedom' might not be broadly understood.

> Response: We thank the reviewer for pointing out this omission. We agree that the gauge transformation is a critical step that requires explicit mathematical and physical justification. To address this, we have added a new subsection to the Appendix titled "Gauge freedom and frequency-decoupling."
>
> This section details the exact algebraic cancellation and maps the gauge invariance to the physical intuition of replicator invariance under shifts in absolute fitness. The core of this new explanation reads (p. 16 lines 2-27).
>
> The full potential $\Psi_g(\mathbf{x})$ and the observable lineage dynamics are invariant under an

allele-independent scalar shift applied to ψ_g, yielding $\psi_g \to \psi_g + \xi(\mathbf{x})$. Because the log-normalization term $\phi(\mathbf{x})$ is determined by the probability conservation constraint, this shift algebraically factors out of the logarithm:

$$\phi(\mathbf{x}) \to \log\left(\sum_g x_g e^{\psi_g + \xi(\mathbf{x})}\right) = \log\left(e^{\xi(\mathbf{x})} \sum_g x_g e^{\psi_g}\right) = \xi(\mathbf{x}) + \phi(\mathbf{x}) \tag{1}$$

Consequently, the arbitrary shift $\xi(\mathbf{x})$ cancels exactly in the difference $\Psi_g(\mathbf{x}) = \psi_g - \phi(\mathbf{x})$. This exact gauge invariance is the time-integrated equivalent of the standard replicator invariance under shifts in absolute fitness, $f_g(\mathbf{x}) \to f_g(\mathbf{x}) + C(\mathbf{x})$. It represents the reproductive-value equivalent of the familiar principle that absolute fitness is unobservable; only relative fitness differences govern observable frequency dynamics.

We use this gauge freedom to minimize the frequency-dependent coupling of ψ_g. Taylor expanding the Ito-drift term gives:

$$\frac{1}{N_e} e^{\psi_g - \phi(\mathbf{x})} = \frac{1}{N_e}\left[1 + \psi_g - \phi(\mathbf{x}) + O\left((\psi_g - \phi(\mathbf{x}))^2\right)\right] \tag{2}$$

Selecting the specific gauge shift $\xi(\mathbf{x}) = \frac{1}{N_e}(1 - \phi(\mathbf{x}))$ absorbs the shared frequency-dependence at linear order. This reduces the g-dependent Ito drift to $\frac{1}{N_e}\psi_g + O\left(\frac{1}{N_e}(\psi_g - \phi(\mathbf{x}))^2\right)$, yielding the first-order decoupled equation:

$$-\partial_t \psi_g(t) = \sum_{g'} \mu_{gg'}\left(e^{\psi_{g'} - \psi_g} - 1\right) + f_g - \frac{1}{N_e}\psi_g \tag{3}$$

This isolates the lineage-specific dynamics to first order, establishing the decoupled system we use for inference.

Reviewer Comment: At the top of p. 6, there's an assumption that $\Psi_g(x) = \psi_g - \phi(x)$, i.e., that the frequency dependence of Ψ can be contained in a separate term ϕ. This assumption is important for the remaining calculations, but it isn't closely examined in the text. What does this mean physically or biologically?

Response: We agree that the physical meaning of this decomposition needed to be stated plainly in the main text. This decomposition was forced by the mathematical requirements. We have revised the text introducing this assumption to clarify that the genotype-specific term ψ_g determines the mutational dynamics, while the shared term $\phi(x)$ absorbs the complex background interactions to enforce the zero-sum normalization constraint of the carrying capacity. The revised text reads (p.3 line 10 - p.4 line 12).

For the lineage dynamics Equation 8 to decouple from population state, the potential differences $\Psi_{g'}(\mathbf{x}) - \Psi_g(\mathbf{x})$ must be independent of $\mathbf{x}$. At the same time, the normalization constraint $\sum_g x_g e^{\Psi_g(\mathbf{x})} = 1$ requires that the individual potentials $\Psi_g(\mathbf{x})$ themselves must remain $\mathbf{x}$-dependent. $\Psi_g(\mathbf{x})$ must therefore take an additively separable form, composed of a genotype-specific term ψ_g that determines the mutational dynamics along the lineage, and a shared log-normalization factor $\phi(\mathbf{x})$. This shared term cancels upon subtraction in Equation 8, but provides the required $\mathbf{x}$-dependence to enforce the normalization constraint:

$$\Psi_g(\mathbf{x}) = \psi_g - \phi(\mathbf{x}) \quad \text{where } \phi(\mathbf{x}) = \log\left(\sum_g x_g e^{\psi_g}\right). \tag{4}$$

Reviewer Comment: One of the interesting aspects of this work is the connection between optimal control and evolutionary theory. There are a number of places where the author describes a control theory interpretation of a selection event. As a metaphor, this is interesting, but I wonder whether this mathematical relationship has deeper consequences. Could the author comment, even hypothetically, on further insights that could be enabled by the parallel between control and population genetics?

Response: We have responded to this request in two ways

First we rewrote the beginning of the discussion how the duality with control theory has informed the derivation. Our results can be seen as reestablishing the structure of control theory solutions from within population genetics. (p.7 line 34)

The derivation of the Hamilton-Jacobi-Bellman (HJB) equation from the lineage distribution relies on viewing Darwinian evolution as a naturally occurring particle filter (see Appendix Control theoretic view of evolving populations). In a particle filter, the target value is read out from the retrospective enrichment of successful lineage states $\log(w_g/x_g)$ [4], this target defines the fitness potential. This algorithmic view justifies the decoupling of the lineage dynamics from the population state $\mathbf{x}$: If Darwinian evolution is a natural particle filter, the targeted dynamics (the optimal control) should be asymptotically independent of the population state. In particle-filter theory, the population state is merely an algorithmic hyperparameter affecting sample efficiency. The derivation of Equation 12 realizes this property, demonstrating that the dynamics of surviving lineages lose their $\mathbf{x}$-dependence in the weak-selection limit.

Identifying the target of the particle filter as an HJB equation yields a dictionary between evolutionary forces and the elements of stochastic control. Fitness f acts as the reward function, the forward mutation rate μ as the uncontrolled dynamics, and the coalescent timescale N_e as the discount factor governing the integration time of the reward. Conditioning descent on survival to time T defines a forward dynamics generated by the biased mutational operator μ^{Ψ}, which corresponds to the generator for the optimal policy. Finally, the fitness potential ψ is the local generating function for this optimal solution—the value function.

Equating lineage paths with optimally controlled trajectories has two immediate consequences. First, it establishes that surviving lineages behave as rational agents optimizing a tradeoff between fitness rewards and the entropic cost of mutational steering. This perspective clarifies the counterintuitive backward-propagating effect of survivorship bias. Second, this equivalence provides a constructive method for building the lineage transition rates. Rather than attempting to integrate the $\mathbf{x}$-dependent Kimura diffusion directly, the Bellman formalism [13] dictates a specific solution sequence: One first solves the HJB equation for the value function (ψ), and then uses this potential to explicitly construct the generator for optimal control (μ^{ψ}). We recover this exact control-theoretic sequence from within population genetics, (Equation 11 and Equation 12). This allows evolutionary inference that avoids the computationally prohibitive high-dimensional integration of direct methods.

Second, we added a subsection to "The landscape of reproductive value," to demonstrate the theoretical consequence of the value function Ψ independent of the likelihood specifically its ability to calculate evolvability trade-offs (p.9 line 66):

Because Ψ integrates parameters such as f, μ, and N_e into a single scalar field, shifts in any structural parameter correspond to a change $\Delta\Psi$. Ψ thus defines a general effective scaled selection coefficient $2\Delta\Psi(\mathbf{x}, t, T) \approx \sigma_{\text{eff}}(\mathbf{x}, t, T)$, establishing a common metric for disparate evolutionary forces. For example, consider the deletion of TP53 during tumor progression. The deletion imposes an immediate replication penalty ($\Delta f < 0$) but in-

creases the variance of μ. The control framework specifies that a net positive σ_{eff} emerges exactly when the altered μ sufficiently reduces the entropic cost of reaching future high-fitness genotypes within a specified timescale $T - t$. The potential Ψ thus calculates the trade-off between instantaneous replication and long-term access to adaptive trajectories, providing a retrospective dual to the "survival of the flattest" phenomenon in quasispecies theory [25, 21, 26] and the selection for evolvability in modifier theory [7, 24, 14].

Furthermore, because the reviewer asked us to comment hypothetically on further insights, we note two other directions opened by this exact mapping that remain active areas of future work:

- The Information Cost of Selection: In stochastic control, the deviation from the uncontrolled dynamics (μ) to the optimal policy (μ^{ψ}) is measured by a Kullback-Leibler (KL) divergence. Hypothetically, this represents the informational or thermodynamic "work" required by selection to localize the population in sequence space. Fully realizing this insight requires determining exactly how much KL cost a finite population can sustain, which could provide a rigorous new bound on the limits of selection.
- Translating Control Algorithms to Phylogenetics: The mapping to the Hamilton-Jacobi-Bellman (HJB) equation allows numerical methods from optimal control, such as reinforcement learning, to address the frequency-dependent case ($\Psi(\mathbf{x})$). However, extending this to phylogenies requires a distinct controller for each lineage. Because these controllers interact via the coalescent hazard, the inference becomes a multi-agent reinforcement learning (MARL) problem where the population $\mathbf{x}$ must be incorporated into the state space. Generating convergent approximations of $\Psi^i(\mathbf{x})$ for each lineage i to construct a valid phylogenetic likelihood remains an open problem.

Reviewer Comment: In the Discussion, the author makes a nice comparison between the approach proposed in the current paper and existing tree- or trajectory-based inference approaches. There, the comment that trajectory-based approaches rely only on marginal allele frequencies does not appear to be entirely correct... by considering the evolution of the full g¨enotype distribution across time, they can address this specific and important concern.

Response: The reviewer is absolutely correct that some trajectory approaches utilize the full population path integral to handle joint distributions. We had used "marginal" incorrectly to refer to marginalizing over the rest of the genome (i.e. ignoring linkage). We have rewritten the section to better describe the difficulties posed by linked selection and partial solutions using empirical covariance matrices. The corrected text reads (p.9 line 40):

For isolated alleles of interest, the associated Kimura propagator can be evaluated directly via spectral expansions or efficient MCMC sampling [22, 9, 15]. However, applying this method to the space of linked alleles renders the full population propagator intractably high-dimensional. Assuming linkage equilibrium restores tractability but discards the covariation required to distinguish true selection from genetic draft. One alternative is to approximate the joint continuous dynamics using a multivariate Gaussian likelihood [8, 3], but this requires the inversion of high-dimensional, ill-conditioned empirical covariance matrices.

References

[1] Mark Ancliff and Jeong-Man Park. Spin coherent state representation of the crow-kimura and eigen models of quasispecies theory. J. Stat. Phys., 143(4):636–656, May 2011.

[2] Ellen Baake and Frederic Alberti. Solving the selection-recombination equation: Ancestral lines under selection and recombination. arXiv [math.PR], March 2020.

[3] Vince Buffalo and Graham Coop. Estimating the genome-wide contribution of selection to temporal allele frequency change. Proc. Natl. Acad. Sci. U. S. A., 117(34):20672–20680, August 2020.

[4] Pierre Del Moral. Feynman-kac formulae: Genealogical and interacting particle systems with applications. Probability and Its Applications (New York). Springer, New York, NY, December 2011.

[5] Odo Diekmann, Pierre-Emanuel Jabin, Stéphane Mischler, and Benoît Perthame. The dynamics of adaptation: an illuminating example and a hamilton-jacobi approach. Theor. Popul. Biol., 67(4):257–271, June 2005.

[6] Esteban Domingo, Julie Sheldon, and Celia Perales. Viral quasispecies evolution. Microbiol. Mol. Biol. Rev., 76(2):159–216, 2012.

[7] Thomas F Hansen. The evolution of genetic architecture. Annu. Rev. Ecol. Evol. Syst., 37(1):123–157, December 2006.

[8] Christopher J R Illingworth, Jayna Raghwani, David Serwadda, Nelson K Sewankambo, Merlin L Robb, Michael A Eller, Andrew R Redd, Thomas C Quinn, and Katrina A Lythgoe. A de novo approach to inferring within-host fitness effects during untreated HIV-1 infection. PLoS Pathog., 16(6):e1008171, June 2020.

[9] Paul A Jenkins and Dario Spanò. Exact simulation of the Wright–Fisher diffusion. aoap, 27(3):1478–1509, June 2017.

[10] Keiji Kanazawa, Daphne Koller, and Stuart Russell. Stochastic simulation algorithms for dynamic probabilistic networks. Proceedings of the Eleventh conference on Uncertainty in artificial intelligence, pages 346–351, 1995.

[11] H J Kappen. Path integrals and symmetry breaking for optimal control theory. J. Stat. Mech., 2005(11):P11011, November 2005.

[12] Hilbert J Kappen. Linear theory for control of nonlinear stochastic systems. Phys. Rev. Lett., 95(20):200201, November 2005.

[13] Donald E Kirk. Optimal Control Theory: An Introduction. Courier Corporation, January 2004.

[14] Michael Lynch. Evolution of the mutation rate. Trends Genet., 26(8):345–352, August 2010.

[15] Cyriel Paris, Bertrand Servin, and Simon Boitard. Inference of selection from genetic time series using various parametric approximations to the wright-fisher model. G3, 9(12):4073–4086, December 2019.

[16] Igor M Rouzine, John Wakeley, and John M Coffin. The solitary wave of asexual evolution. Proc. Natl. Acad. Sci. U. S. A., 100(2):587–592, January 2003.

[17] Hans-Christian Ruiz and Hilbert J Kappen. Particle smoothing for hidden diffusion processes: Adaptive path integral smoother. IEEE Trans. Signal Process., 65(12):3191–3203, June 2017.

[18] David B Saakian. A new method for the solution of models of biological evolution: Derivation of exact steady-state distributions. J. Stat. Phys., 128(3):781–798, July 2007.

[19] David B Saakian and Chin-Kun Hu. Solution of classical evolutionary models in the limit when the diffusion approximation breaks down. Phys. Rev. E., 94(4-1):042422, October 2016.

[20] David B Saakian, Jin Ming Koh, and Kang Hao Cheong. Approximate perturbative solutions of quasispecies model with recombination. Phys. Rev. E., 99(6-1):062407, June 2019.

[21] Josep Sardanyés, Santiago F Elena, and Ricard V Solé. Simple quasispecies models for the survival-of-the-flattest effect: The role of space. J. Theor. Biol., 250(3):560–568, February 2008.

[22] Joshua G Schraiber, Steven N Evans, and Montgomery Slatkin. Bayesian inference of natural selection from allele frequency time series. Genetics, 203(1):493–511, May 2016.

[23] Lee Van Valen. A new evolutionary law. Evolutionary theory, 1:1–30, 1973.

[24] Günter P Wagner and Lee Altenberg. Perspective: Complex adaptations and the evolution of evolvability. Evolution, 50(3):967–976, June 1996.

[25] C O Wilke, J L Wang, C Ofria, R E Lenski, and C Adami. Evolution of digital organisms at high mutation rates leads to survival of the flattest. Nature, 412(6844):331–333, July 2001.

[26] Claus O Wilke. Quasispecies theory in the context of population genetics. BMC Evol. Biol., 5:44, August 2005.

April 29, 2026
RE: GENETICS-2026-309292

Dr. Colin LaMont
Oslo universitetssykehus Radiumhospitalet
Department of Cancer Immunology
Oslo University Hospital, Montebello
Oslo 0310
Norway

Dear Dr. LaMont:

Congratulations, your manuscript titled "Optimal Control and the Dynamics of Ancestral Lineages" is accepted for publication in GENETICS! Many thanks for submitting your research to the journal.

To Proceed to Publication:
1. Format your article according to GENETICS style: https://academic.oup.com/genetics/pages/author-guidelines

2. Ensure that you comply with data and community resource citation guidelines:
https://academic.oup.com/genetics/pages/author-guidelines#section-5-9-2

3. Upload your final files at https://genetics.msubmit.net

4. Add oupsupport@scipris.com and genetics.oup@novatechset.com (or the domains @scipris.com and @novatechset.com) to your email program's "safe senders" list. You will be contacted by both at various points during the production process.

Notes:
- Your currently-accepted manuscript (unedited, as submitted, reviewed, and accepted) will be published at GENETICS and deposited into PubMed as an Advance Access article. Notify sourcefiles@thegsajournals.org before signing your license if you do not wish to publish your article via Advance Access.

- We invite you to submit an original color figure related to your paper for consideration as cover art. Please email your submission to the editorial office or upload it with your final files. You can submit a small-sized image for evaluation, and if selected, the final image must be a TIFF file 2513px wide by 3263px high (8.375 by 10.875 inches; resolution of 600ppi). Please avoid graphs and small type.

- After files are sent to Oxford University Press we use SciPris to manage article licensing and payment. If you do not have a SciPris account, you will receive an email from no-reply@scipris.com to sign up to use Oxford University Press' author portal. After logging in, follow the online instructions to sign your license and arrange any payment due.

If you have any questions or encounter any problems while uploading your accepted manuscript files, please email the editorial office at sourcefiles@thegsajournals.org.

Sincerely,

Kavita Jain
Associate Editor
GENETICS

Approved by:
Nick Barton
Senior Editor
GENETICS

Review comments (if applicable):

Reviewer #1 :

I thank the author for thorough revisions that have improved the clarity of the paper, and I have no additional comments.
